# Two protons, two positrons, and four electrons: Covalent bond with van der Waals characteristics

**DOI:** 10.1126/sciadv.aej0387

**Published:** 2026-09-30

**Authors:** Jorge Charry, Alexandre Tkatchenko

**Affiliations:** ^1^ Department of Physics and Materials Science, University of Luxembourg, L-1511 Luxembourg City, Luxembourg.; ^2^ Luxembourg Researchers Hub asbl, 223 rue de Luxembourg, L-4222 Esch-sur-Alzette, Luxembourg.; ^3^ HPC Platform, University of Luxembourg, L-4364 Esch-sur-Alzette, Luxembourg.

## Abstract

Classifying interactions is key in the physical sciences, and bonding mechanisms in matter-antimatter systems remain particularly enigmatic. Here, we focus on a paradigmatic example of positronium hydride (PsH) dimer composed of two protons, two positrons, and four electrons, whose bonding nature has been previously described as either ionic, covalent, or van der Waals like. Accurate quantum Monte Carlo calculations show that the two positrons occupy a delocalized molecular orbital that envelopes the two hydrogen anions and responds as a collective dipole to an applied electric field. This positronic bonding stems from quantum correlations that resemble a single covalent bond formed between negatively charged pseudonuclei, but with a bond strength commensurate with the traditional van der Waals interaction. Our findings suggest that the ability to form delocalized proto-bonds is a more general property of quantum systems and could be present in a broader class of particles, antiparticles, and quasiparticles interacting with matter.

## INTRODUCTION

Understanding the interaction of positrons with matter and fields is important for a wide range of scales, from cosmological to subatomic. The range of outstanding puzzles involving positrons includes the mechanism for asymmetry between matter and antimatter in the early universe (*1*) to the computation of electron-positron field polarization in quantum electrodynamics (*2*). At the atomic scale, the detection of gamma rays produced during the electron-positron pair annihilation process is the basis for useful technological applications, such as positron emission tomography (PET) to map biological activity in tissues (*3*) and positron annihilation lifetime spectroscopy to probe the (im)purity of materials (*4*, *5*).

More recently, positrons entered molecular sciences as well. Low-energy positrons (*6*, *7*) can form stable states with electrons, atoms, and molecules before the pair annihilation process. Experimental detection via the vibrational Feshbach resonance technique has confirmed positron binding in roughly 100 molecules, with observed binding energies reaching 300 meV (*8*). These molecular-positron complexes have attracted notable interest because their relatively long lifetimes (on the order of nanosecond) allow them to interact with nuclear motion in molecules, which typically unfolds on femtosecond to picosecond timescales (*9*). As a result, positrons can influence or alter molecular reaction pathways. Furthermore, because annihilation rates are intrinsically linked to the molecular environment, new positronium imaging techniques have emerged to exploit this sensitivity (*10*), extracting more biological and medically relevant information within living tissues that complements standard PET.

Therefore, to discover and understand novel positronic chemistry, robust theoretical tools are needed to accurately model interacting matter-antimatter systems. A critical challenge is to properly account for electron-positron quantum correlation effects (*11*–*13*). In recent decades, most theoretical studies have focused on the binding of positrons to stable molecules (*7*, *9*, *11*, *12*, *14*–*19*). It was also found that positrons can stabilize the interaction between two or more otherwise repelling atoms or ions, forming a previously unidentified type of bond rather than simply attaching to a molecule. Those systems range from the case of one positron between two hydrogen anions H^−^e^+^H^−^ (*20*), to other single positron bonds in halides (*21*, *22*), beryllium (*13*, *23*) and molecular anion dimers (*24*), the case of two positrons in two hydrides (*25*), halides (*26*), three hydride centers (*27*), and even three-positron two hydride system (*28*).

One of the simplest paradigmatic cases of the proton-electron-positron–containing molecule is positronium hydride (PsH). Its ground state consists of hydrogen anion dressed with a positron in its outer shell. Because PsH is a neutral moiety, it has been proposed that the interaction between two PsH molecules is purely noncovalent (*29*, *30*). However, Bressanini (*25*) used quantum Monte Carlo (QMC) calculations to demonstrate that, at an H-H distance of 6.0 bohr, the two positrons in the PsH dimer bind two otherwise mutually repulsive hydrogen anions with an interaction energy of 6.5 kcal/mol. This is similar to the single-positron case, which, however, binds two hydrogen anions by 14.8 kcal/mol (*20*). It is thus clear that the nature of unique bonding in the PsH dimer is unsolved (*25*, *31*, *32*), shifting from covalent as in H_2_ or Li_2_, ionic like as in *q*^−^2*q*^+^*q*^−^ arrangement model of three constituents with a partial charge *q*, back to a “super van der Waals” description driven by strong quantum correlations, all with unique features compared with regular electronic bonds.

A simplified scheme for these different proposed mechanisms is shown in Fig. 1. Ultimately, these overlapping yet distinct features indicate a bonding mechanism that defies standard classification, leaving the true character of the bond an open puzzle. We propose that this situation arises because the traditional chemical bond analysis tools based on particle densities and bond strengths are insufficient to explain interactions in matter-antimatter systems. Instead, we offer a solution by performing converged and quantitative QMC calculations and analyzing the polarization response properties of the PsH dimer. QMC is particularly suited for this task as it allows for the integration of explicitly correlated wave functions (*12*–*14*, *33*–*37*), which are essential for accurately capturing the complex correlation between positrons and molecular systems. By computing (PsH)_2_ polarizability as a function of the internuclear distance and applying the polarizability decomposition analysis recently introduced in reference (*38*), we aim to provide a comprehensive analysis on the characterization of bonding in (PsH)_2_. We start by deconstructing the traditional bonding mechanisms.

**Fig. 1.**
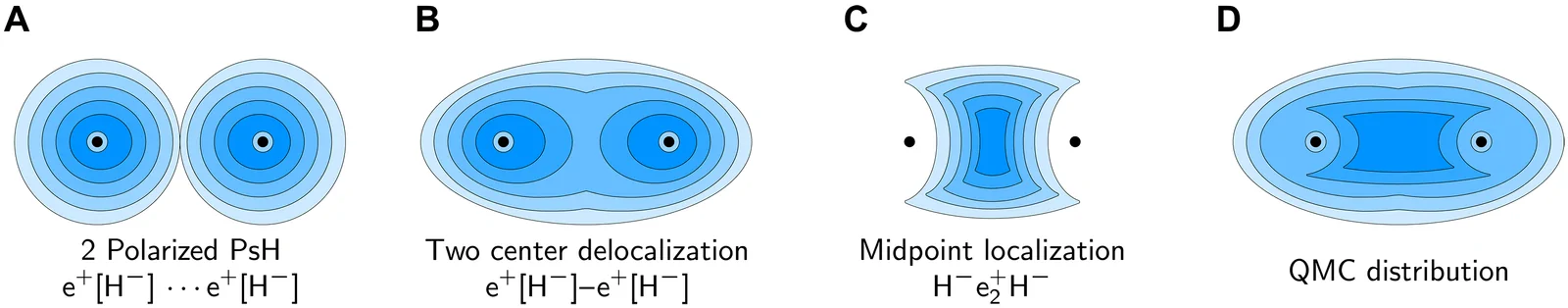
Schematic comparison for possible bond mechanism interpretations in (PsH)_2_. Simplified contour distribution for the positronic density distribution in (PsH)_2_. (**A**) Two isolated PsH polarizing each other and interacting via van der Waals mechanism. (**B**) Expected distribution from electronic covalent bond analogues. (**C**) Localized *q*^−^2*q*^+^*q*^−^ ionic-like distribution. (**D**) QMC positronic density distribution at the equilibrium geometry.

## RESULTS

### (PsH)_2_: Not a traditional chemical bond

The interaction in (PsH)_2_ and other positron bonds has been previously analyzed by the following approaches: (i) comparing the properties of its potential energy curve against regular electronic systems; (ii) analyzing the topology of the electronic and positronic density; and (iii) performing energy decompositions commonly expressed in terms of electrostatic, polarization, exchange, and correlation components.

Although H_2_ is the standard model for chemical bonding, positron-bonded systems share features closer to alkali metals, specifically in their internuclear distances and bond strengths, although their underlying physics is different (*20*, *21*, *25*–*27*, *31*).

Given the above similarities, the bonding densities for electronic and positronic systems have also been compared and analyzed in detail (*25*, *31*), shown here in Fig. 2 as two-dimensional (2D) density plots (and its Laplacian in the Supplementary Materials) and in Fig. 3 as evolution of 1D density slices as a function of the internuclear distance. As reported before, by comparing against the densities of two noninteracting PsH, the electronic density in (PsH)_2_ remains largely unaffected upon the addition of the positron (*31*). On the other hand, the positron pair seems to serve as the primary mediator of the bond since its major charge density redistribution from one PsH to the dimer. Although the positronic distribution surrounding the two H^−^ anions mimics the appearance of a covalent bond, this is insufficient to prove a traditional covalent character due to several unique topological features compared with those of H_2_ and Li_2_. Specifically, the positronic distribution exhibits a “hole” at the nuclear positions instead of the standard cusp, a result of the electrostatic repulsion between the positrons and the nuclei. Moreover, the density is mostly localized in the middle of the two ions, reaching a maximum along the internuclear axis and also along the perpendicular plane. Despite the statistical noise, the corresponding positronic Laplacian density profile shows a clear trend toward accumulation regions at the midpoint between the two H^−^ units, with depletion near the nuclei due to the positron repulsion as reported before (*31*) (see also in fig. S1). As seen in Fig. 3, at large distances, the positronic density exhibits two distinct maxima per PsH, localized around each hydride center, with their corresponding minima at the nucleus position due to the positron-nuclei repulsion. As the fragments approach the equilibrium distance, the two internuclear maxima fuse into a single prominent maximum at the bond midpoint. This topology mirrors the emergence of a “pseudoatom” or non-nuclear attractors (NNAs) bridging the two centers, characteristic of loosely bound, highly polarizable quantum particles. A feature present in molecular electrides (*39*, *40*) and in Li_2_ at a much lower density scale, but absent in H_2_. Due to the high electrostatic attractive interaction, the concentration of positive charge (2*q*^+^) symmetrically localized between the two negatively charged hydrogen anion centers (*q*^−^) might initially suggest a localized ionic-like *q*^−^2*q*^+^*q*^−^ arrangement. Not to be confused with the ionic bond *q*^−^*q*^+^ in standard electronic molecular systems. However, energy decomposition analysis (*31*, *32*) indicated that such attraction is insufficient, instead the stabilization is mainly due to electron-positron and positron-positron correlation effects. Because correlation-driven interactions between neutrally charged atoms are commonly associated with dispersion interactions, Goli *et al.* (*32*) compared the binding energy of (PsH)_2_ with the dispersion energy estimated from previously computed van der Waals *C*_6_ and *C*_8_ coefficients of isolated PsH (*30*) as $E_{\text{disp}}^{\left(\text{PsH}-\text{PsH}\right)}\approx -\frac{C_{6}}{R^{6}}-\frac{C_{8}}{R^{8}}$. For PsH the ratio of dispersion coefficients is relatively high, $C_{8}/C_{6}\approx 90$, which is comparable to heavy alkali (Na-Na: 74/Cs-Cs: 149) or alkaline earth metals (Sr-Sr: 68/Ba-Ba: 91), in contrast to lower ratios within first and second period (H-H: 19/Li-Li: 60) or noble gases (He-He: 10/Xe-Xe: 42). As a consequence, at the equilibrium distance, $E_{\text{disp}}^{\left(\text{PsH}-\text{PsH}\right)}=-12.9$ kcal/mol roughly captures the order of magnitude of the DMC binding energy of −6.5 kcal/mol, but with a 70% contribution from *C*_8_ and 30% from *C*_6_ (see the Supplementary Materials). Therefore, given the relatively high binding energy compared with regular electronic van der Waals interactions (1 to 4 kcal/mol), the prominent role of positron-positron correlation, and the large dispersion coefficients, it was suggested to denote the bond as a super van der Waals bond (*32*).

**Fig. 2.**
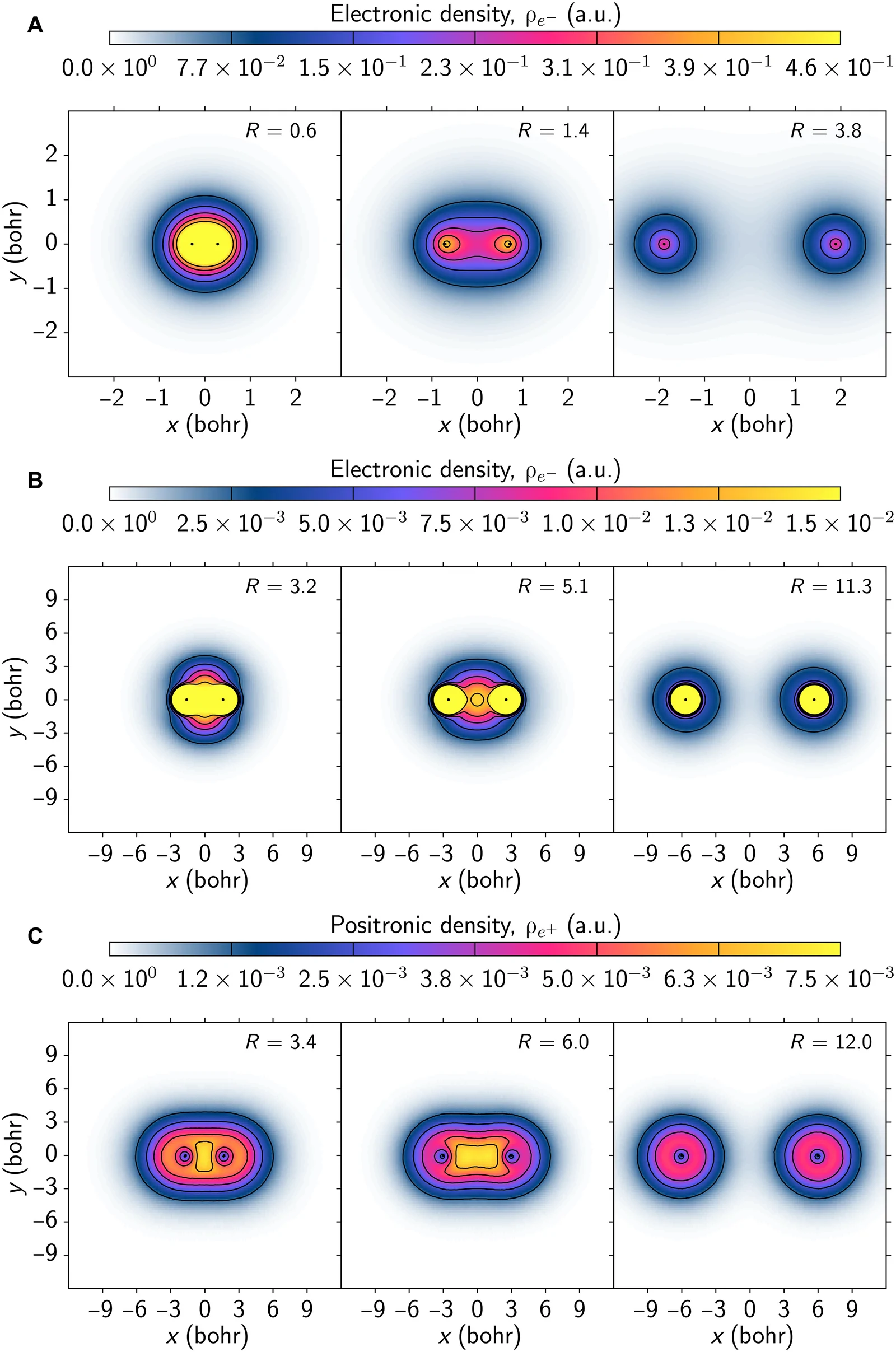
Comparison of bonding densities in diatomic molecules. CCSD natural orbital electronic density for H_2_ (**A**) and Li_2_ (**B**). DMC Positronic density for (PsH)_2_ (**C**). Each panel—left, middle, and right—corresponds to electronic or positronic densities computed at three representative internuclear separations: short range [near the dissociation channel Ps_2_ + H_2_ for (PsH)_2_], equilibrium distance, and dissociation limit.

**Fig. 3.**
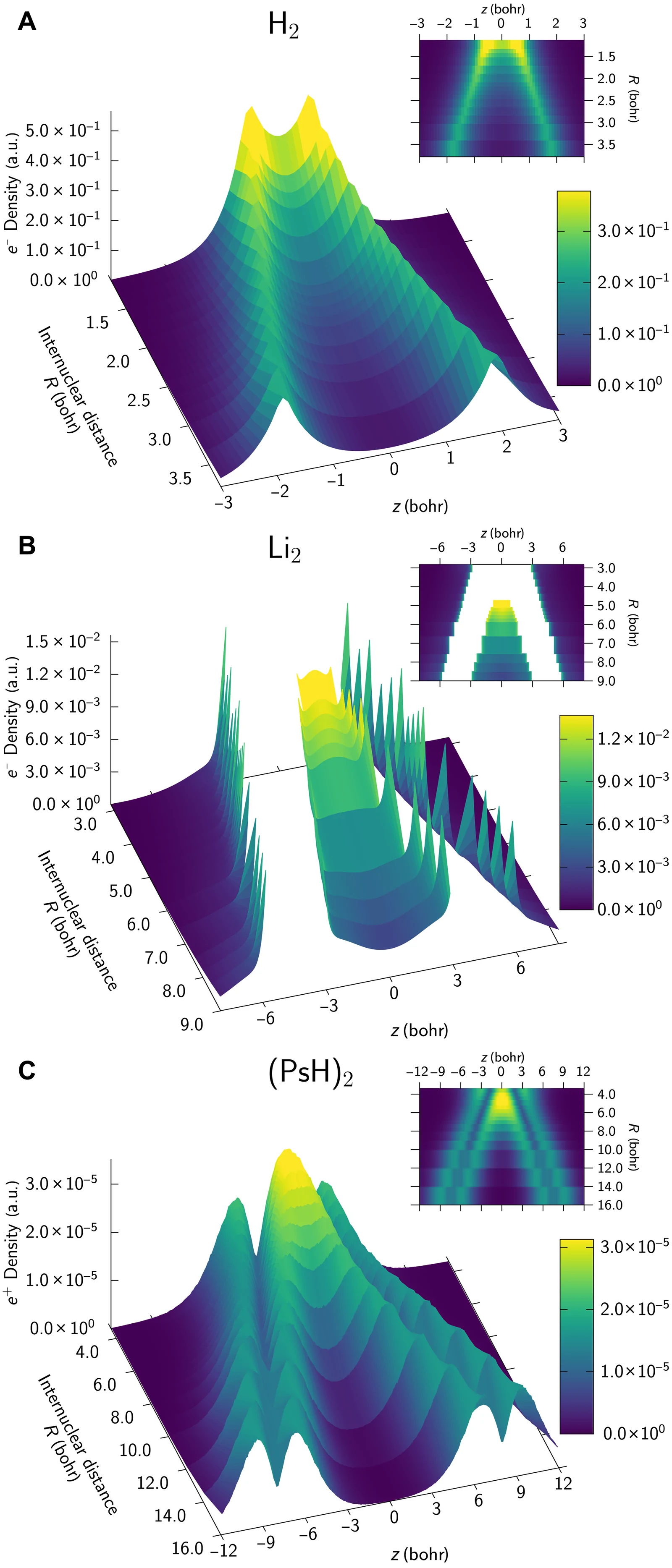
One-dimensional bonding density evolution. Interpolated surfaces of density slices on the *xy* plane parallel to the bond axis (*z* axis), showing their evolution with respect to the internuclear distance. CCSD natural orbital electronic density for H_2_ (**A**) and Li_2_ (**B**). DMC Positronic density for (PsH)_2_ (**C**).

### (PsH)_2_: Not a van der Waals bond

The first argument against a van der Waals bond is that this interpretation contradicts the large reorganization of the positronic density from PsH to (PsH)_2_ (Figs. 2 and 3). This opposes the picture of two isolated atoms interacting through instantaneous dipoles required for a van der Waals dispersion interaction. In addition, the DMC energy curve decays slower than the van der Waals $R^{-6}$ potential.

To quantify particle sharing, we computed in Fig. 4 the delocalization indices (DIs) (*41*–*43*) using a multicomponent compacted active-space full configuration interaction (FCI) approach (*20*, *44*, *45*), validated against our QMC potential energy surface (see the Supplementary Materials). Around the equilibrium distance, the positronic DI is notably larger than its electronic counterpart and exhibits a characteristic sigmoid decay upon dissociation, a characteristic of shared, covalent interactions analogous to Li_2_ and H_2_ (*38*, *46*). Conversely, the electronic DI is roughly half the magnitude and decays exponentially, indicating a nonbonded character (*46*) and confirming that the positronic cloud acts as the primary collective glue in the dimer. A broader study across a wider array of multipositronic complexes remains an open avenue to fully map these unique DI topologies.

**Fig. 4.**
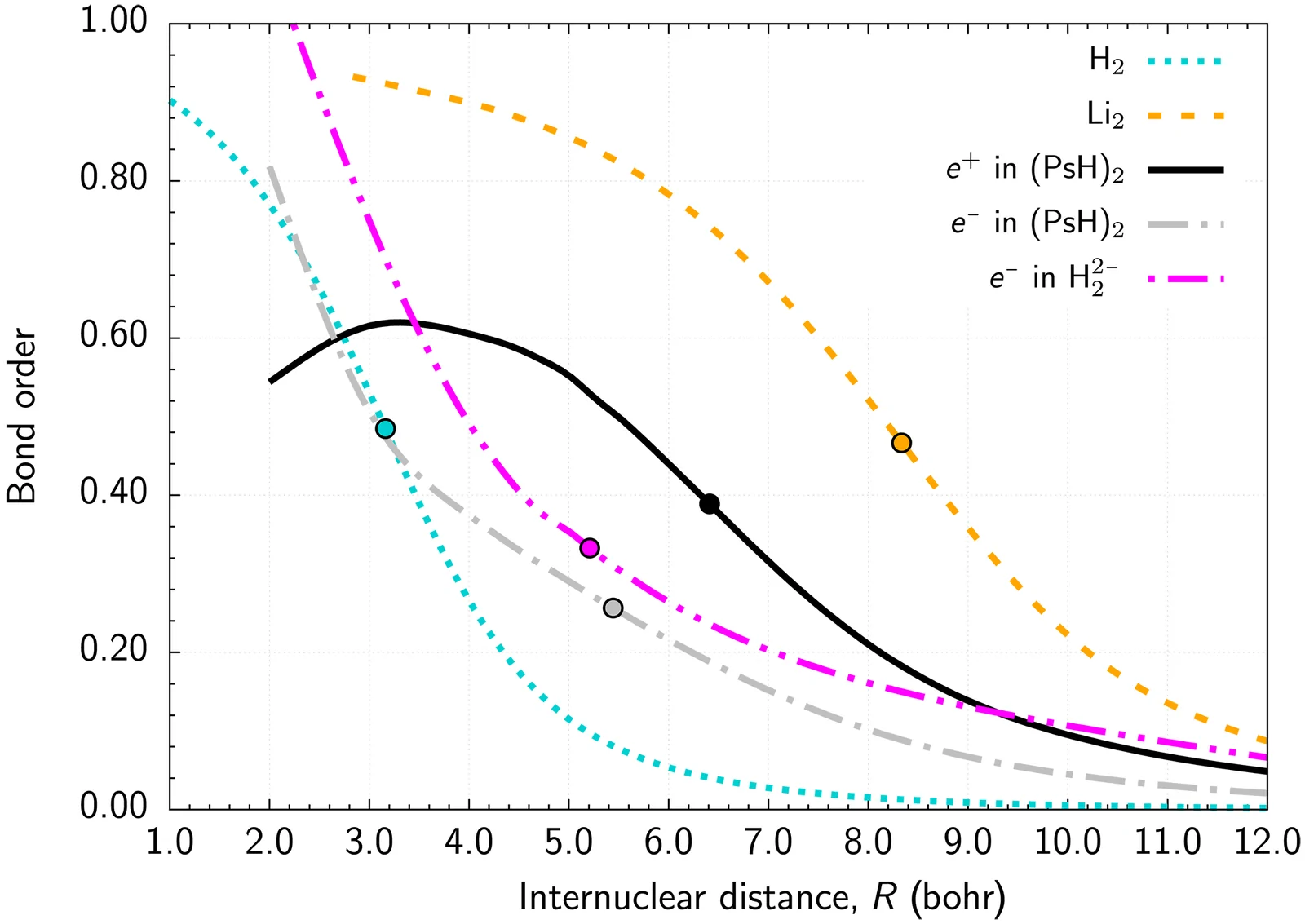
Evolution of electronic and positronic bond orders across internuclear distances. Delocalization index (DI) bond order for H_2_, Li_2_, H^2–^_2_, and positronic and electronic DI in (PsH)_2_. The data for H_2_ and Li_2_ were taken from the open Zenodo repository of (*38*). Circles are used to highlight the identification of an inflexion point.

As additional evidence, we present in Fig. 5 the evolution of the energy and relative polarizability along the internuclear distance of (PsH)_2_. We also include data from H_2_ and Li_2_, systems previously used for comparison with positronic bonds (*47*, *38*). As seen from there, the strength of the (PsH)_2_ bond is close to the Li_2_ case, which is considered as a weak covalent bond (*48*). The polarizability values were derived from both the first-order derivative of the dipole moment and the second-order derivatives of the energy (refer to the Supplementary Materials for details on our methodology). Our results show that the polarizability [130.5 (2) bohr^3^], computed along the axis connecting the hydrogen nuclei, is considerably higher than the sum of two noninteracting PsH monomers (84.6 bohr^3^). This enhancement indicates a substantial redistribution of the wave function in comparison with that of the isolated monomers. This behavior mirrors the polarizability trends observed in covalently bonded diatomic molecules at their equilibrium distances (*38*, *47*, *49*–*51*).

**Fig. 5.**
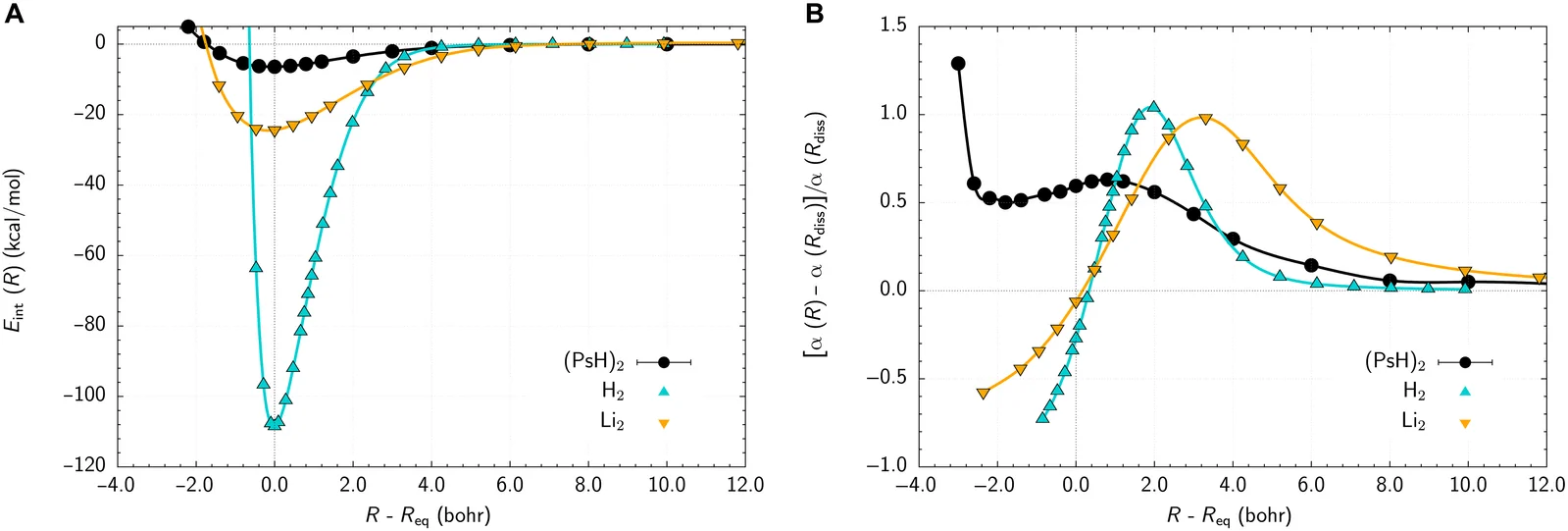
Interaction energies and polarizabilities curves for diatomic molecules. (**A**) Interaction energies [*E*_AB_(*R*) − *E*_A_ − *E*_B_] and (**B**) relative polarizability (parallel component with respect to the bond axis) changes as a function of the internuclear separation shifted to the equilibrium for PsH_2_, H_2_, and Li_2_.

At shorter distances, when the system factorizes in the sum of Ps_2_ and H_2_ (*25*), the polarizability abruptly increases, as expected, but this regime is not the focus of this study.

To shed further light on the characteristics of the polarizability, we decompose the dipole moment operator into electronic $\mu^{e}=-\sum_{i=1}^{N_{e}}r_{i}$ and positronic $\mu^{p}=\sum_{i=1}^{N_{p}}r_{i}$ parts, allowing to compute separate polarizability contributions of the two leptonic particles

$$\begin{array}{cc} \alpha_{\mathrm{ab}} & ={\left.\frac{\partial \mu_{a}^{e}\left(F_{b}\right)}{\partial F_{b}}\right|}_{F_{b}=0}+{\left.\frac{\partial \mu_{a}^{p}\left(F_{b}\right)}{\partial F_{b}}\right|}_{F_{b}=0}\\ & =\alpha_{\mathrm{ab}}^{e}+\alpha_{\mathrm{ab}}^{p}a,b\in \left[x,y,z\right] \end{array}$$ (1)

The black curves in Fig. 6 (A and B) show the contribution of positronic and electronic polarizability, respectively. At all internuclear separations of (PsH)_2_, the largest contribution comes from the delocalized positrons, while the electronic response is almost negligible. These results suggest that, from the point of view of its response properties, a PsH system can be interpreted as a positronic cloud that fully screens the electronic response of the H^−^ anion it surrounds. An isolated H^−^ anion exhibits a large polarizability of 203 a.u. (*29*), which stems from loosely bound electrons that can easily be distorted. This observation contradicts the arguments of Yan (*30*), who proposed a simple estimate of the polarizabilities of PsH (42.3 a.u.) as a weakly bound state between Ps and an H atom (40.5 = 36 + 4.5 a.u.), and confirms later studies based on the wave functions distributions, which conclude that the structure of PsH is that of an H anion slightly perturbed by the positron (*52*). The screened H^−^ anion behavior seen in PsH is also visible in (PsH)_2_ at all distances.

**Fig. 6.**
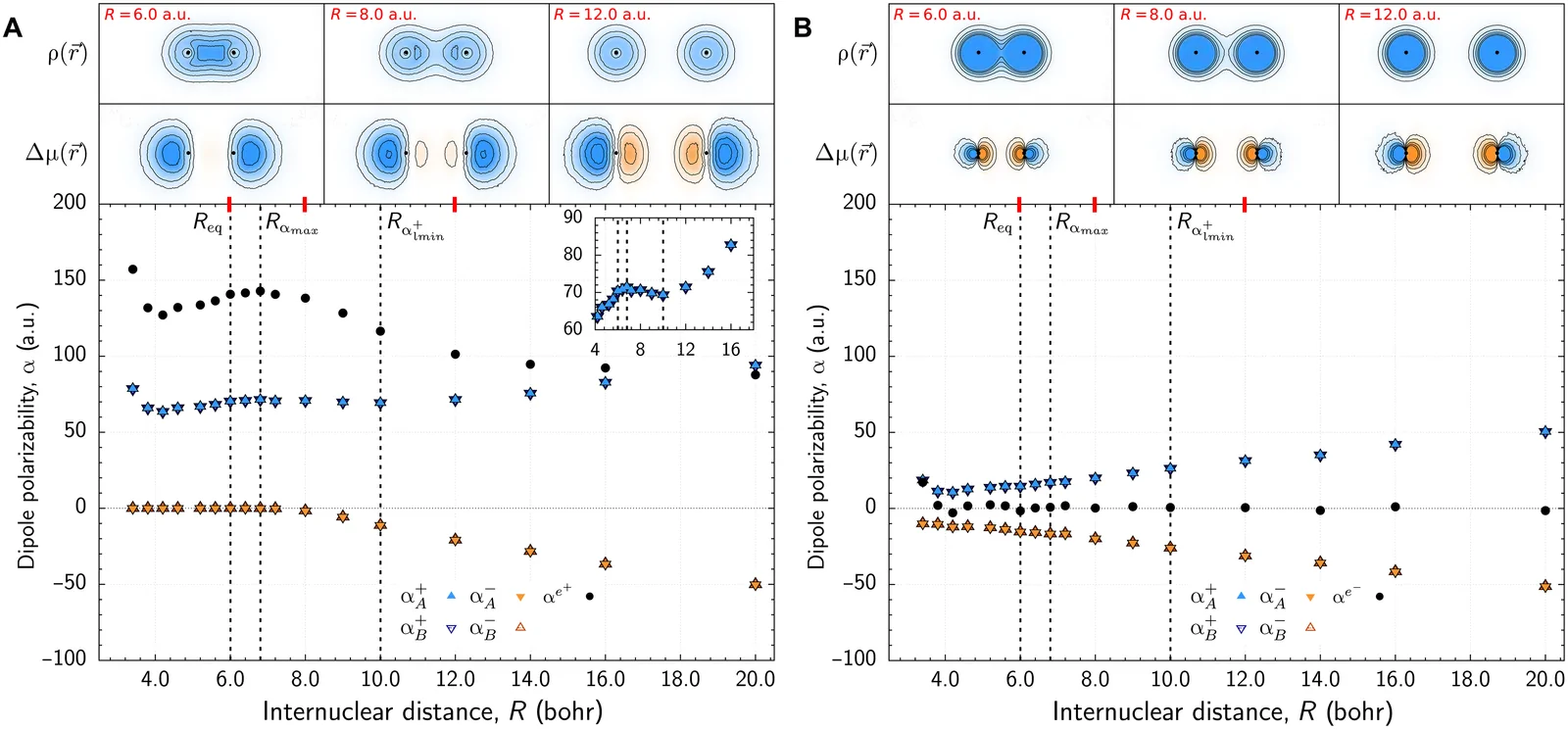
Dipole response under an applied electric field on PsH_2_. Spatial decomposition curves of positronic (**A**) and electronic (**B**) dipole polarizabilities according to Eq. 2, as illustrated in the top panels for the 2D local density ρ and dipole variation Δμ plots at representative internuclear separations. Orange and blue regions correspond to a decrease and an increase of positronic or electronic local quantities, respectively. Dotted vertical lines correspond to equilibrium distance $R_{\text{eq}}$, maximum polarizability peak $R_{\alpha_{\text{max}}}$, and local minima of $\alpha_{\text{lmin}}^{+}$ polarizability component $R_{\alpha_{\text{lmin}}^{+}}$. The (A) inset shows a zoomed-in region near the critical points on $\alpha^{+}$ curves.

To better understand the origin of the polarizability changes, we used the local decomposition proposed by us in (*38*), which consists in computing the local polarizabilities of the two fragments *A* and *B* that participate in the bond, separating them into an accumulation (+) and a depletion (−) region, such that

$$\alpha_{\mathrm{zz}}=\alpha_{A}^{+}+\alpha_{A}^{-}+\alpha_{B}^{+}+\alpha_{B}^{-}$$ (2)

where

$$\left\{ \begin{array}{c} \alpha_{\Omega }^{+}=\int_{V_{\Omega }}\text{max}\left\{{\left.0,r_{z}\frac{\partial \rho \left(\vec{r},F_{z}\right)}{\partial F_{z}}\right|}_{F_{z}=0}\right\}d\vec{r}\\ \alpha_{\Omega }^{-}=\int_{V_{\Omega }}\text{min}\left\{{\left.0,r_{z}\frac{\partial \rho \left(\vec{r},F_{z}\right)}{\partial F_{z}}\right|}_{F_{z}=0}\right\}d\vec{r} \end{array}\right.$$ (3)

with Ω∈[A,B] and assuming that the *z* axis corresponds with the direction of the bond. Note that, while the polarizability decomposition used here is fundamentally origin dependent, a feature discussed in detail in our previous work (*38*), this does not introduce any ambiguity for (PsH)_2_ due to its spatial symmetry. In essence, the bond center is uniquely defined as the exact geometric midpoint, which simultaneously coincides with the center of mass, the center of charge, and the topological bond critical point. On the other hand, this introduces a linear dependency with the internuclear systems, which allows for interpreting the different bonded, bond-breaking, and dissociation regions under the context of the midpoint origin dependency.

Figure 6 shows the above decomposition for the local variations of the positronic (A) and electronic (B) polarizabilities along the (PsH)_2_ bond. In the panels above, we also include a positronic density contour map and a local dipole variation at representative distances for an easier interpretation.

An analysis of the individual accumulation and depletion components around fragments *A* and *B* reveals a distinct pattern. The local electronic polarizability components, denoted as $\alpha_{A}^{+},\alpha_{B}^{+}$ for accumulation and $\alpha_{A}^{-},\alpha_{B}^{-}$ for depletion, scale linearly with the internuclear distance (Fig. 6B), due to the formation of two dipoles in opposite directions that cancel out, leading to an almost zero total electronic dipole polarizability. This behavior mirrors a “pseudo” noncovalent interaction, suggesting that the electrons remain localized around their respective nuclei with no considerable exchange between the two anions (*38*). Therefore, the unperturbed electronic response to an external field, in combination with the electronic static nature upon the addition of positrons, reveals that electrons are not the bond mediators.

In contrast, the positronic polarizability components exhibit a behavior characteristic of traditional covalent bonds (Fig. 6A) (*38*). At the equilibrium distance of 6.0 bohr, an external electric field induces positronic density accumulation on the side of one nucleus facing away from the other, with a specular effect occurring at the opposite anion. Together, these local shifts establish a molecular dipole between hydrogen anions that evolves as a function of the internuclear distance. As the fragments separate, this molecular dipole increases until the polarizability reaches its maximum ($\alpha_{\text{max}}$) at 7.2 bohr. Beyond this peak, the dipole in the extrabond regions ($\alpha_{B}^{+},\alpha_{A}^{+}$) begins to diminish. Similarly, density reorganization in the intrabond region generates an opposing dipole ($\alpha_{B}^{-},\alpha_{A}^{-}$) that decreases linearly. A final transition occurs at 10 bohr, marked by local minima in $\alpha_{B}^{+}$ and $\alpha_{A}^{+}$. This threshold identifies the onset of the dissociation region, where $\alpha_{B}^{+}$ and $\alpha_{A}^{+}$ resume a linear increase, similar to the trend seen in the intrabond region. In this regime, the electric field induces two collinear atomic dipoles, one at each hydrogen nucleus, signaling the dissociation limit where the dimer’s polarizability converges to the sum of two noninteracting PsH fragments.

### (PsH)_2_: Proto-bond with van der Waals strength

The leading-order positronic polarizability curve of the PsH dimer closely mirrors that of a weak electronic covalent bond, as observed in typical diatomic systems (*47*, *38*). In contrast, the much smaller electronic response remains largely static, effectively screened by the positronic cloud. From this perspective, this system is best described as a positronic distribution surrounding relatively unperturbed H^−^ anions. This similarity in the local dipole response is particularly remarkable given the unique positronic density distribution of (PsH)_2_, which is highly localized at the bond’s midpoint, a feature that might superficially suggest an ionic-like bond *q*^−^2*q*^+^*q*^−^ character, but the underlying physics tells a different story. Such density distribution suggests an intriguing parallel to NNAs in molecular electrides (*39*, *40*); this connection could inspire the design and exploration of more positron-bonded compounds.

The covalent-like behavior arises because the positronic pair mediating the bond occupies a shared single “molecular orbital” that also encapsulates the two negatively charged hydrogen atoms, responding as a single molecular dipole to an external electric field. In this framework, the two H^−^ anions act as “pseudonuclei” with diffused electronic wave functions that remain essentially separated from one another. This picture illustrates the unique features of the bond and contradicts the long-held and still discussed assumption that the PsH dimer bonds purely through enhanced van der Waals interactions, despite the relatively low binding energy of 6.5 kcal/mol compared with standard covalent bonds. Moreover, our results establish polarizability as a vital complement to density analysis, offering a comprehensive framework for characterizing chemical bonds.

## DISCUSSION

In conclusion, we have systematically evaluated the nature of the bonding interaction in the (PsH)_2_ by contextualizing our findings with existing perspectives from the literature. A consistent picture that (PsH)_2_ is not a traditional chemical bond emerges by analyzing a diverse set of physical and chemical descriptors, spanning energetics, energy decomposition, electronic and positronic density topologies, and their Laplacian and evolution along the bond axis.

The electron-pair and positron-pair sharing through DIs coupled with our analysis of electronic and positronic polarizabilities response firmly demonstrate that the (PsH)_2_ is not a van der Waals bond but rather is characterized by a delocalized (proto)-bond with distinct covalent signatures compared with traditional electronic covalent interaction, defying traditional classification. Yet, the low binding energy of 6.5 kcal/mol is closer in magnitude to the typical scale of 1 to 4 kcal/mol for van der Waals interaction, estimated from its dispersion coefficients, given the large polarizability of each PsH monomer. Considering that, we lastly conclude that (PsH)_2_ is a proto-bond with van der Waals strength.

Overall, the tools and analysis presented in this work provide a comprehensive understanding of intramolecular positronic interactions, which are gradually reshaping and expanding the community’s perspective on the field of positronic chemistry. This work not only resolves the historical ambiguity surrounding this exotic system but also establishes a rigorous framework for diagnosing nonclassical bonding in quantum matter.

These findings broadens the scope of bonding topologies of quantum systems, paving the way for the exploration of unconventional bond-like chemistry in a broader class of particles, antiparticles, and quasiparticles interacting with matter, for example, muon $\mu^{-}$ in muon-catalyzed fusion (*53*), antimuon $\mu^{+}$ in vibrational bonding for $\text{Br}\mu^{+}\text{Br}$ like compounds (*54*), or electron-hole pairs in materials.

## MATERIALS AND METHODS

The polarizability and electronic density for H_2_ and Li_2_ were taken from (*38*), having been originally computed with Psi4 code (*55*) at the CCSD(T) and CCSD levels, respectively, using the aug-cc-pVTZ basis set. The CC density was generated from the natural orbitals of the CCSD ground-state one-electron reduced density matrix using the Molden interface of the Psi4 code. The multicomponent FCI (FCI/aug-cc-pVTZ) was computed from a reduced active space selected from the natural orbitals of a preceding CISD calculation using the FCI implementation of the openLOWDIN code (*20*, *45*) and Jadamilu library for the large sparse matrix diagonalization (*44*).

The detailed description of the QMC methods and wave function applied for this investigation is reported in the Supplementary Materials. A single Slater determinant was used for the electronic wave functions, built as a linear combination of atomic-centered Gaussian-type orbitals, and the positronic part of the wave function is always built through the EPO ansatz generalized in (*34*). To properly describe all correlations in these systems, we have also introduced a novel Jastrow described in the Supplementary Materials. For all the calculations, we apply a modified version of the QMeCha QMC package (*37*).

The H atomic basis is built with (3s1p)/[1s1p] contracted Gaussian orbitals. For simplicity, the same set of orbitals is used for both the positronic and fermionic wave determinantal parts. The dynamical Jastrow basis is built from (3s2p) uncontracted and not normalized GTOs centered on each atom and from (3s2p) uncontracted electron-positron GTOs. All the parameters of the wave function are optimized simultaneously, including the contracted exponents and coefficients, the molecular orbital coefficients, the cusps parameters, and the linear and orbital parameters of the dynamical Jastrow factor. Regarding the DMC calculations, each optimized VMC wave function was used as the guiding function, and the estimation of the DMC energy was performed with 6400 walkers divided into 5000 blocks, each 1000 steps long, for a time step of 0.001 hartree^−1^.

The QMC density plots were obtained by counting the number of particles of each weighted configuration in a 3D grid of 101,101,401 points with a length step of 0.02, 0.02, and 0.01 bohr. For all the electronic properties that do not commute with the Hamiltonian, the second-order estimator (SOE) was used, for which ${\langle \hat{A}\rangle }_{\text{SOE}}\approx 2{\langle \hat{A}\rangle }_{\text{DMC}}-{\langle \hat{A}\rangle }_{\text{VMC}}$. All response properties are computed through a polynomial fit using fields of variable strength in the interval [0.001, 0.007] (see the Supplementary Materials for further details).

## Supplementary Materials

### This PDF file includes:

Supplementary Text Figs. S1 to S17 Tables S1 to S3 References

## References

[1] D. Bödeker, W. Buchmüller, Baryogenesis from the weak scale to the grand unification scale. Rev. Mod. Phys. 93, 035004 (2021).

[2] A. Tkatchenko, D. V. Fedorov, Casimir self-interaction energy density of quantum electrodynamic fields. Phys. Rev. Lett. 130, 041601 (2023). 10.1103/PhysRevLett.130.041601 36763430

[3] S. D. Bass, S. Mariazzi, P. Moskal, E. Stepien, *Colloquium*: Positronium physics and biomedical applications. Rev. Mod. Phys. 95, 021002 (2023).

[4] D. W. Gidley, H.-G. Peng, R. S. Vallery, Positron annihilation as a method to characterize porous materials. Annu. Rev. Mater. Res. 36, 49–79 (2006).

[5] A. N. Singh, Positron annihilation spectroscopy in tomorrow’s material defect studies. Appl. Spectrosc. Rev. 51, 359–378 (2016).

[6] C. D. Anderson, The positive electron. Phys. Rev 43, 491–494 (1933).

[7] S. Schippers, E. Sokell, F. Aumayr, H. Sadeghpour, K. Ueda, I. Bray, K. Bartschat, A. Murray, J. Tennyson, A. Dorn, M. Yamazaki, M. Takahashi, N. Mason, O. Novotný, A. Wolf, L. Sanche, M. Centurion, Y. Yamazaki, G. Laricchia, C. M. Surko, J. Sullivan, G. Gribakin, D. W. Savin, Y. Ralchenko, R. Hoekstra, G. O’Sullivan, Roadmap on photonic, electronic and atomic collision physics: II. Electron and antimatter interactions. J. Phys. B 52, 171002 (2019).

[8] J. R. Danielson, E. Arthur-Baidoo, C. M. Surko, Improved positron-molecule binding energies and estimations using molecular parameters. Phys. Rev. A 111, 042809 (2025).

[9] A. Dupasquier, Allen P. Mills, Roberto S. Brusa, Eds., *Physics with Many Positrons* (IOS Press, 2010).

[10] P. Moskal, K. Dulski, N. Chug, C. Curceanu, E. Czerwiński, M. Dadgar, J. Gajewski, A. Gajos, G. Grudzień, B. C. Hiesmayr, K. Kacprzak, Ł. Kapłon, H. Karimi, K. Klimaszewski, G. Korcyl, P. Kowalski, T. Kozik, N. Krawczyk, W. Krzemień, E. Kubicz, P. Małczak, S. Niedźwiecki, M. Pawlik-Niedźwiecka, M. Pędziwiatr, L. Raczyński, J. Raj, A. Ruciński, S. Sharma, R. Y. S. Shivani, M. Silarski, M. Skurzok, E. Ł. Stępień, M. Szczepanek, F. Tayefi, W. Wiślicki, Positronium imaging with the novel multiphoton PET scanner. Sci. Adv. 7, eabh4394 (2021). 10.1126/sciadv.abh4394 PMC11559468 34644101

[11] J. Hofierka, B. Cunningham, C. M. Rawlins, C. H. Patterson, D. G. Green, Many-body theory of positron binding to polyatomic molecules. Nature 606, 688–693 (2022). 10.1038/s41586-022-04703-3 PMC9217750 35732760

[12] G. Cassella, W. M. C. Foulkes, D. Pfau, J. S. Spencer, Neural network variational Monte Carlo for positronic chemistry. Nat. Commun. 15, 5214 (2024). 10.1038/s41467-024-49290-1 PMC11189582 38890287

[13] S. Upadhyay, A. Benali, K. D. Jordan, Capturing correlation effects in positron binding to atoms and molecules. J. Chem. Theory Comput. 20, 9879–9893 (2024). 10.1021/acs.jctc.4c00727 PMC11603616 39288307

[14] D. Bressanini, M. Mella, G. Morosi, Positronium chemistry by quantum Monte Carlo. I. Positronium-first row atom complexes. J. Chem. Phys. 108, 4756–4760 (1998).

[15] M. Charlton, J. W. Humberston, *Positron Physics* (Cambridge Univ. Press, 2009).

[16] Y. C. Jean, P. E. Mallon, D. M. Schrader, *Principles and Applications of Positron and Positronium Chemistry* (World Scientific, 2003).

[17] H. Chojnacki, K. Strasburger, “Quantum chemical calculations on positronic systems,” in *Explicitly Correlated Wave Functions in Chemistry and Physics* (Springer, 2003), pp. 439–463.

[18] G. F. Gribakin, J. A. Young, C. M. Surko, Positron-molecule interactions: Resonant attachment, annihilation, and bound states. Rev. Mod. Phys. 82, 2557–2607 (2010).

[19] Y. Kita, M. Tachikawa, “Quantum Monte Carlo study of the binding of a positron to polar molecules,” in *Advances in Quantum Monte Carlo* (American Chemical Society, 2012), pp. 157–173.

[20] J. Charry, M. T. N. Varella, A. Reyes, Binding matter with antimatter: The covalent positron bond. Angew. Chem. Int. Ed. Engl. 57, 8859–8864 (2018). 10.1002/anie.201800914 29770555

[21] F. Moncada, L. Pedraza-González, J. Charry, M. T. d. N. Varella, A. Reyes, Covalent bonds in positron dihalides. Chem. Sci. 11, 44–52 (2020). 10.1039/c9sc04433g PMC7472554 32953003

[22] T. Tachibana, D. Hoshi, Y. Nagashima, Molecular ion desorption from LiF (110) surfaces by positron annihilation. Phys. Rev. Lett. 131, 143201 (2023). 10.1103/PhysRevLett.131.143201 37862658

[23] R. Porras-Roldan, J. Charry, F. Moncada, R. Flores-Moreno, M. T. d. N. Varella, A. Reyes, Watch out electrons!: Positron binding redefines chemical bonding in Be_2_. Chem. Sci. 16, 22322–22332 (2025). 10.1039/d5sc05711f PMC12628274 41268515

[24] J. P. Cassidy, J. Hofierka, B. Cunningham, D. G. Green, Many-body theory calculations of positronic-bonded molecular dianions. J. Chem. Phys. 160, 084304 (2024). 10.1063/5.0188719 38407288

[25] D. Bressanini, Two positrons can form a chemical bond in (PsH)2. J. Chem. Phys. 155, 054306 (2021). 10.1063/5.0059721 34364352

[26] D. Archila-peña, F. Moncada, J. Charry, T. N. Varella, R. Flores-moreno, F. J. Torres, A. Reyes, Two-positron-bonded dihalides: Ps_2_XY (X, Y = F, Cl, Br). Chem. Eur. J. 30, 202402618 (2024). 10.1002/chem.202402618 39259931

[27] J. Charry, F. Moncada, M. Barborini, L. Pedraza-González, M. T. d. N. Varella, A. Tkatchenko, A. Reyes, The three-center two-positron bond. Chem. Sci. 13, 13795–13802 (2022). 10.1039/d2sc04630j PMC9710307 36544737

[28] D. Bressanini, e^+^(PsH)_2_: A three-positron molecule with a positronic chemical bond. J. Chem. Phys. 156, 154302 (2022). 10.1063/5.0089157 35459325

[29] M. Mella, D. Bressanini, G. Morosi, Variational Monte Carlo calculation of dynamic multipole polarizabilities and van der Waals coefficients of the PsH system. Phys. Rev. A 63, 024503 (2001).

[30] Z.-C. Yan, Polarizabilities and dispersion coefficients of positronium hydride. J. Phys. B At. Mol. Opt. Phys. 35, L345–L349 (2002).

[31] M. Goli, D. Bressanini, S. Shahbazian, On the nature of the two-positron bond: Evidence for a novel bond type. Phys. Chem. Chem. Phys. 25, 29531–29547 (2023). 10.1039/d3cp03003b 37905569

[32] M. Goli, D. Bressanini, S. Shahbazian, The two-positron gluic bond as a manifestation of ‘super’ van der Waals interactions. Phys. Chem. Chem. Phys 28, 11154–11160 (2026). 10.1039/d6cp00453a 42017993

[33] Y. Kita, M. Tachikawa, N. D. Drummond, R. J. Needs, A variational monte carlo study of positronic compounds using inhomogeneous backflow transformations. Chem. Lett. 39, 1136–1137 (2010).

[34] J. A. Charry Martinez, M. Barborini, A. Tkatchenko, Correlated wave functions for electron–positron interactions in atoms and molecules. J. Chem. Theory Comput. 18, 2267–2280 (2022). 10.1021/acs.jctc.1c01193 PMC9009097 35333513

[35] F. Marsusi, E. Mostaani, N. D. Drummond, Quantum Monte Carlo study of three-dimensional Coulomb complexes: Trions and biexcitons, hydrogen molecules and ions, helium hydride cations, and positronic and muonic complexes. Phys. Rev. A 106, 62822 (2022).

[36] K. A. Simula, J. E. Muff, I. Makkonen, N. D. Drummond, Quantum Monte Carlo study of positron lifetimes in solids. Phys. Rev. Lett. 129, 166403 (2022). 10.1103/PhysRevLett.129.166403 36306750

[37] M. Barborini, J. Charry, M. Ditte, A. Leventis, G. Kafanas, A. Tkatchenko, QMeCha: Quantum Monte Carlo package for fermions in embedding environments. J. Chem. Phys. 164, 062501 (2026). 10.1063/5.0311994 41677286

[38] J. Charry, M. Barborini, A. Tkatchenko, Unraveling chemical bonding mechanisms through dipole moment variations under external electric fields. Phys. Chem. Chem. Phys. 27, 23044–23055 (2025). 10.1039/d5cp02953h 41098001

[39] V. Postils, M. Garcia-Borràs, M. Solà, J. M. Luis, E. Matito, On the existence and characterization of molecular electrides. Chem. Commun. 51, 4865–4868 (2015). 10.1039/c5cc00215j 25697671

[40] S. P. Sitkiewicz, E. Ramos-Cordoba, J. M. Luis, E. Matito, How many electrons does a molecular electride hold? J. Phys. Chem. A 125, 4819–4835 (2021). 10.1021/acs.jpca.1c02760 34038110

[41] J. G. Ángyán, E. Rosta, P. R. Surján, Covalent bond orders and atomic valences from correlated wavefunctions. Chem. Phys. Lett. 299, 1–8 (1999).

[42] C. Outeiral, M. A. Vincent, Á. M. Pendás, P. L. A. Popelier, Revitalizing the concept of bond order through delocalization measures in real space. Chem. Sci. 9, 5517–5529 (2018). 10.1039/c8sc01338a PMC6049528 30061983

[43] A. F. Silva, L. J. Duarte, P. L. Popelier, Contributions of IQA electron correlation in understanding the chemical bond and non-covalent interactions. Struct. Chem. 31, 507–519 (2020).

[44] M. Bollhöfer, Y. Notay, JADAMILU: A software code for computing selected eigenvalues of large sparse symmetric matrices. Comput. Phys. Commun. 177, 951–964 (2007).

[45] R. Flores-Moreno, E. Posada, F. Moncada, J. Romero, J. Charry, M. Díaz-Tinoco, S. A. González, N. F. Aguirre, A. Reyes, LOWDIN: The any particle molecular orbital code. Int. J. Quantum Chem. 114, 50–56 (2014).

[46] M. García-Revilla, P. L. Popelier, E. Francisco, Á. Martín Pendás, Nature of chemical interactions from the profiles of electron delocalization indices. J. Chem. Theory Comput. 7, 1704–1711 (2011). 10.1021/ct2001842 26596434

[47] D. Hait, M. Head-Gordon, When is a bond broken? The polarizability perspective. Angew. Chem. Int. Ed. Engl. 62, e202312078 (2023). 10.1002/anie.202312078 37713599

[48] A. J. Sterling, D. S. Levine, A. Aldossary, M. Head-Gordon, Chemical bonding and the role of node-induced electron confinement. J. Am. Chem. Soc. 146, 9532–9543 (2024). 10.1021/jacs.3c10633 38532619

[49] J. Rychlewski, An accurate calculation of the polarizability of the hydrogen molecule and its dependence on rotation, vibration and isotopic substitution. Mol. Phys. 41, 833–842 (1980).

[50] G. Gopakumar, M. Abe, M. Hada, M. Kajita, Dipole polarizability of alkali-metal (Na, K, Rb)-alkaline-earth-metal (Ca, Sr) polar molecules: Prospects for alignment. J. Chem. Phys 140, 224303 (2014). 10.1063/1.4881396 24929384

[51] E. Miliordos, K. L. Hunt, Dependence of the multipole moments, static polarizabilities, and static hyperpolarizabilities of the hydrogen molecule on the H-H separation in the ground singlet state. J. Chem. Phys. 149, 234103 (2018). 10.1063/1.5066308 30579318

[52] D. Bressanini, G. Morosi, Compact boundary-condition-determined wave function for positronium hydride (PsH). J. Chem. Phys 119, 7037–7042 (2003).

[53] Y. Toyama, T. Azuma, D. A. Bennett, W. B. Doriese, M. S. Durkin, J. W. Fowler, J. D. Gard, T. Hashimoto, R. Hayakawa, Y. Ichinohe, K. Ishida, S. Kanda, N. Kawamura, Y. Kino, R. Konishi, Y. Miyake, K. M. Morgan, R. Nakashima, H. Natori, H. Noda, G. C. O’Neil, S. Okada, T. Okumura, K. Okutsu, C. D. Reintsema, K. Sasaki, T. Sato, D. R. Schmidt, K. Shimomura, P. Strasser, D. S. Swetz, T. Takahashi, M. Tampo, H. Tatsuno, J. N. Ullom, I. Umegaki, S. Watanabe, S. Yamada, T. Yamashita, Direct observation of muonic molecules in resonance states critical to muon catalyzed fusion. Sci. Adv. 12, eaed3321 (2026). 10.1126/sciadv.aed3321 PMC13082326 41984964

[54] D. G. Fleming, J. Manz, K. Sato, T. Takayanagi, Fundamental change in the nature of chemical bonding by isotopic substitution. Angew. Chem., Int. Ed. Engl. 53, 13706–13709 (2014). 10.1002/anie.201408211 25303174

[55] D. G. A. Smith, L. A. Burns, A. C. Simmonett, R. M. Parrish, M. C. Schieber, R. Galvelis, P. Kraus, H. Kruse, R. Di Remigio, A. Alenaizan, A. M. James, S. Lehtola, J. P. Misiewicz, M. Scheurer, R. A. Shaw, J. B. Schriber, Y. Xie, Z. L. Glick, D. A. Sirianni, J. S. O’Brien, J. M. Waldrop, A. Kumar, E. G. Hohenstein, B. P. Pritchard, B. R. Brooks, H. F. Schaefer III, A. Y. Sokolov, K. Patkowski, A. E. DePrince III, U. Bozkaya, R. A. King, F. A. Evangelista, J. M. Turney, T. D. Crawford, C. D. Sherrill, Psi4 1.4: Open-source software for high-throughput quantum chemistry. J. Chem. Phys. 152, 184108 (2020). 10.1063/5.0006002 PMC7228781 32414239

[56] S. Varrette, P. Bouvry, H. Cartiaux, F. Georgatos, Management of an academic HPC Cluster: The UL experience, in *Proceedings of the 2014 International Conference on High Performance Computing and Simulation (HPCS 2014)* (IEEE, 2014), pp. 959–967.

[57] J. Jiang, J. Mitroy, Y. Cheng, M. W. J. Bromley, Effective oscillator strength distributions of spherically symmetric atoms for calculating polarizabilities and long-range atom–atom interactions. At. Data Nucl. Data Tables 101, 158–186 (2015).

[58] W. M. C. Foulkes, L. Mitas, R. J. Needs, G. Rajagopal, Quantum Monte Carlo simulations of solids. Rev. Mod. Phys 73, 33–83 (2001).

[59] M. H. Kalos, P. A. Whitlock, “Quantum Monte Carlo,” in *Monte Carlo Methods* (John Wiley & Sons, Ltd., 2000), pp. 159–178.

[60] F. Becca, S. Sorella, *Quantum Monte Carlo Approaches for Correlated Systems* (Cambridge Univ. Press, 2017).

[61] S. Sorella, Generalized Lanczos algorithm for variational quantum Monte Carlo. Phys. Rev. B. 64, 024512 (2001).

[62] S. Sorella, Wave function optimization in the variational Monte Carlo method. Phys. Rev. B. 71, 241103 (2005).

[63] C. Filippi, C. Umrigar, Correlated sampling in quantum Monte Carlo: A route to forces. Phys. Rev. B 61, R16291–R16294 (2000).

[64] P. J. Reynolds, D. M. Ceperley, B. J. Alder, W. A. Lester, Fixed-node quantum Monte Carlo for molecules. J. Chem. Phys. 77, 5593–5603 (1982).

[65] I. Kosztin, B. Faber, K. Schulten, Introduction to the diffusion Monte Carlo method. Am. J. Phys. 64, 633–644 (1996).

[66] M. P. Nightingale, C. J. Umrigar, *Quantum Monte Carlo Methods in Physics and Chemistry*, vol. 525 of *NATO Science Series C* (Kluwer Academic Publishers, published in cooperation with NATO Scientific Affairs Division, 1999).

[67] C. J. Umrigar, M. P. Nightingale, K. J. Runge, A diffusion Monte Carlo algorithm with very small time-step errors. J. Chem. Phys. 99, 2865–2890 (1993).

[68] T. A. Anderson, M. C. Per, C. J. Umrigar, Reducing the time-step errors in diffusion Monte Carlo. J. Chem. Phys. 160, 104110 (2024). 10.1063/5.0190346 38482870

[69] F. Della Pia, B. X. Shi, Y. S. Al-Hamdani, D. Alfé, T. A. Anderson, M. Barborini, A. Benali, M. Casula, N. D. Drummond, M. Dubecký, C. Filippi, P. R. C. Kent, J. T. Krogel, P. López Ríos, A. Lüchow, Y. Luo, A. Michaelides, L. Mitas, K. Nakano, R. J. Needs, M. C. Per, A. Scemama, J. Schultze, R. Shinde, E. Slootman, S. Sorella, A. Tkatchenko, M. Towler, C. J. Umrigar, L. K. Wagner, W. A. Wheeler, H. Zhou, A. Zen, Reproducibility of fixed-node diffusion Monte Carlo across diverse community codes: The case of water–methane dimer. J. Chem. Phys. 163, 104110 (2025). 10.1063/5.0272974 40919967

[70] M. D. Towler, The quantum Monte Carlo method. Phys. Status Solidi B 243, 2573–2598 (2006).

[71] A. D. Buckingham, Permanent and induced molecular moments and long-range intermolecular forces. Adv. Chem. Phys 12, 107–142 (1967).

[72] D. Bressanini, M. Mella, G. Morosi, Stability and positron annihilation of positronium hydride *L* = 0,1,2 states: A quantum Monte Carlo study. Phys. Rev. A 57, 1678–1685 (1998).

[73] M. Casula, C. Attaccalite, S. Sorella, Correlated geminal wave function for molecules: An efficient resonating valence bond approach. J. Chem. Phys. 121, 7110–7126 (2004). 10.1063/1.1794632 15473777

[74] S. F. Boys, N. C. Handy, J. W. Linnett, A calculation for the energies and wavefunctions for states of neon with full electronic correlation accuracy. Proc. Math. Phys. Eng. Sci. 310, 63–78 (1969).

[75] N. D. Drummond, M. D. Towler, R. J. Needs, Jastrow correlation factor for atoms, molecules, and solids. Phys. Rev. B 70, 235119 (2004).

[76] D. Bressanini, The stability of e^+^(H^–^)_2_. J. Chem. Phys. 154, 224306 (2021). 10.1063/5.0053998 34241201

[77] C. Swalina, M. V. Pak, S. Hammes-Schiffer, Analysis of electron-positron wavefunctions in the nuclear-electronic orbital framework. J. Chem. Phys. 136, 164105 (2012). 10.1063/1.4704124 22559468

[78] J. Tiihonen, I. Kylänpää, T. T. Rantala, Computation of dynamic polarizabilities and van der waals coefficients from path-integral monte carlo. J. Chem. Theory Comput. 14, 5750–5763 (2018). 10.1021/acs.jctc.8b00859 30278124

[79] R. A. Ferrell, Theory of positron annihilation in solids. Rev. Mod. Phys. 28, 308–337 (1956).

[80] N. Jiang, D. M. Schrader, Diffusion quantum Monte Carlo calculation of the binding energy and annihilation rate of positronium hydride, PsH. J. Chem. Phys. 109, 9430–9433 (1998).

[81] J. Mitroy, M. W. J. Bromley, G. G. Ryzhikh, “Positronic atoms,” in *New Directions in Antimatter Chemistry and Physics*, C. M. Surko, F. A. Gianturco, Eds. (Kluwer Academic Publishers, 2001), pp. 199–221.

[82] S. Bubin, L. Adamowicz, Nonrelativistic variational calculations of the positronium molecule and the positronium hydride. Phys. Rev. A 74, 052502 (2006).

[83] C. Le Sech, B. Silvi, Study of positronium hydride with a simple wavefunction: Application to the Stark effect of PsH. Chem. Phys. 236, 77–85 (1998).

