# Supplementary material for: Two protons, two positrons, and four electrons: Covalent bond with van der Waals characteristics
Source: Sci Adv. 2026 Sep 30;12(40):eaej0387. doi: 10.1126/sciadv.aej0387 (PMC13626055; doi:10.1126/sciadv.aej0387)
Supplement: Supplementary file 1 [file sciadv.aej0387_sm.pdf]

Science Advances
AAAS

Supplementary Materials for

Two protons, two positrons, and four electrons: Covalent bond with van der Waals characteristics

Jorge Charry and Alexandre Tkatchenko

Corresponding author: Jorge Charry, jorge.charry@researchers-hub.lu;
Alexandre Tkatchenko, alexandre.tkatchenko@uni.lu

Sci. Adv. **12**, eaej0387 (2026)
DOI: 10.1126/sciadv.aej0387

This PDF file includes:

Supplementary Text
Figs. S1 to S17
Tables S1 to S3
References

1 Laplacian of the positronic density

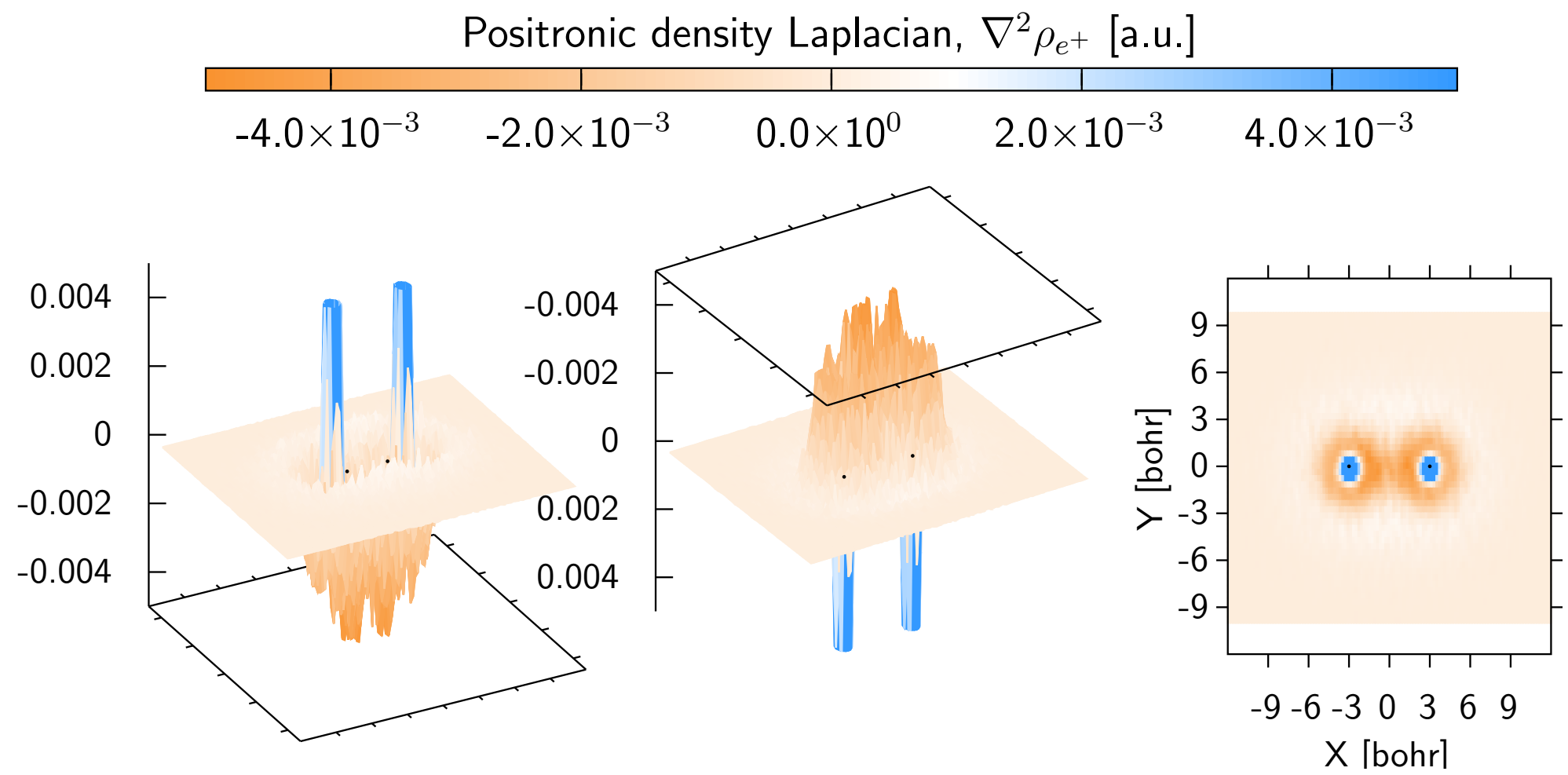

Figure S1: **Laplacian of the QMC positronic density in $(PsH)_2$.**

Three different angle views plots: lateral view (left), inverted lateral (center), and top view (right). The QMC 3D density grid was first smoothed via a Gaussian filtering post-processing, then the Laplacian was estimated using three-point finite differences.

2 Dispersion energy of two non-covalent PsH

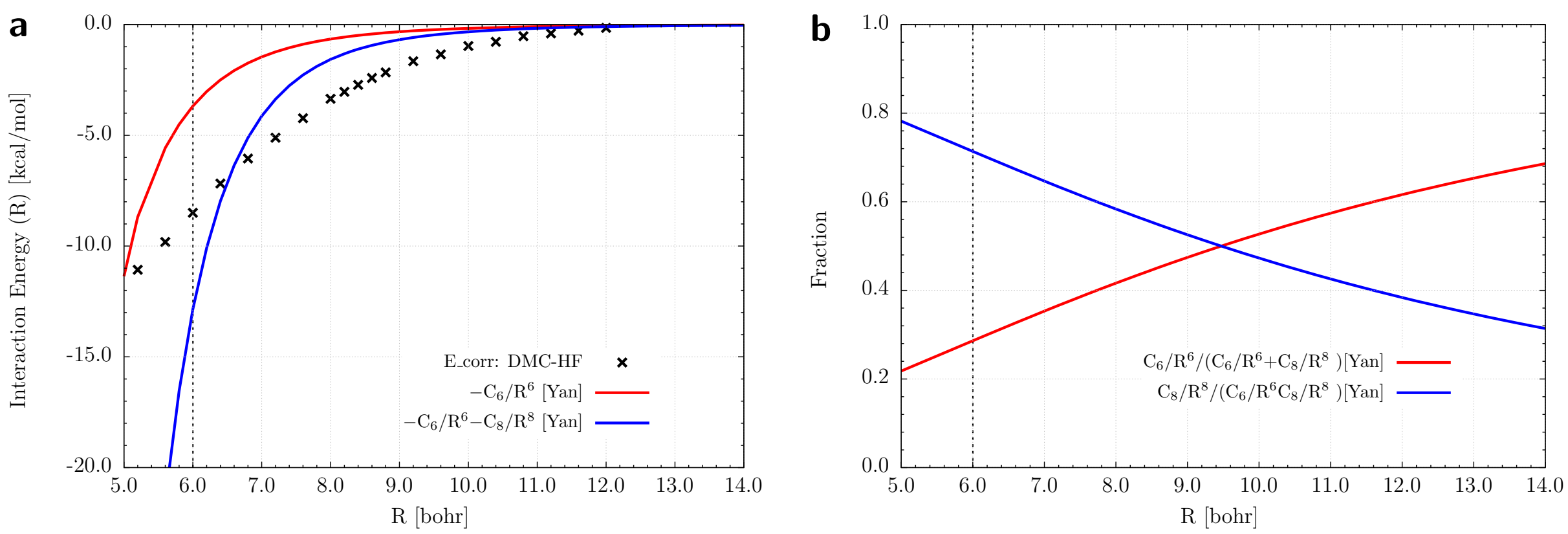

Figure S2: **Dispersion interaction analysis in** $(PsH)_2$**.**

(**a**) Comparison of estimated dispersion energy for PsH-PsH interaction and correlation energy computed from $(PsH)_2$. (**b**) Fraction of C_6 and C_8 terms with respect to the total estimated dispersion energy. Dispersion coefficients C_6 and C_8 taken from Yan et al. (*30*). DMC and HF data from Goli et al (*32*). Dotted vertical line marks the equilibrium distance of $(PsH)_2$ Correlation energy defined as the difference between DMC and HF/aug-cc-pVQZ energies $E_{\mathrm{corr}} = E_{\mathrm{DMC}} - E_{\mathrm{HF}}$

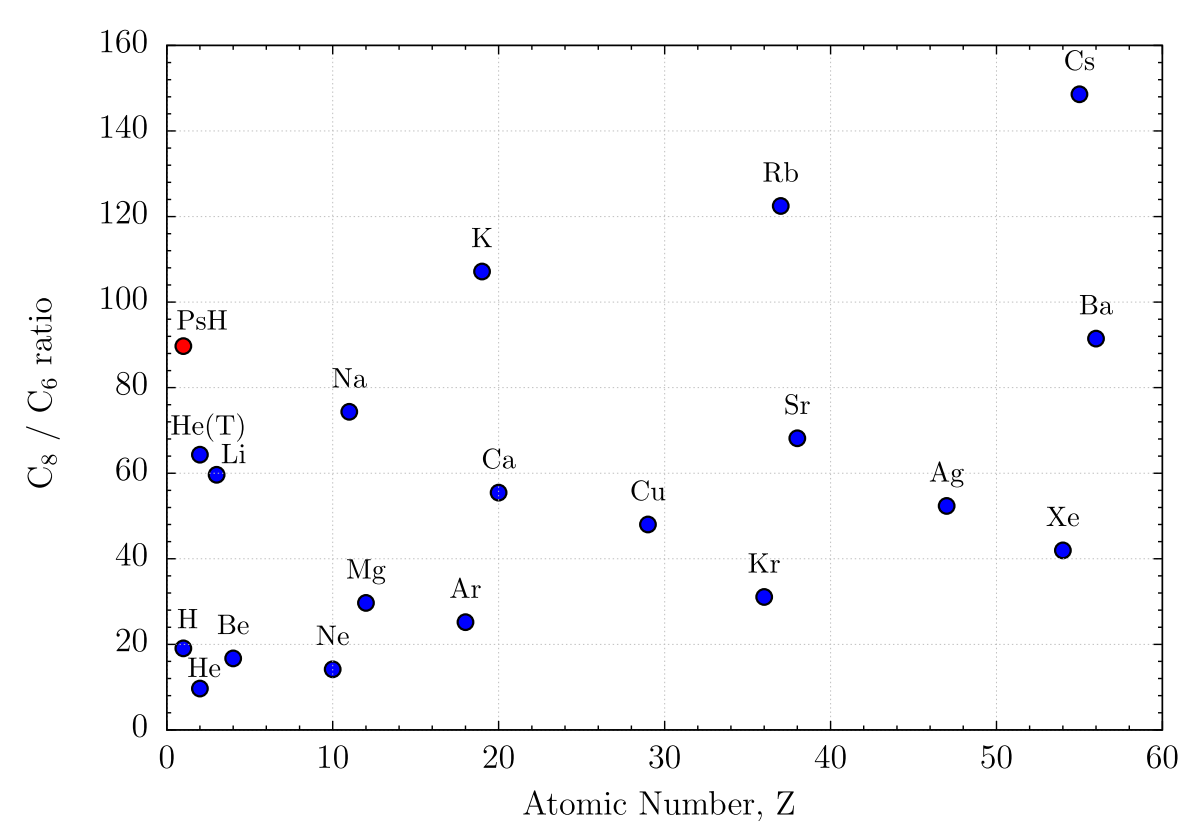

Figure S3: **Comparison of dispersion coefficient ratios in the periodic table of atomic elements**

Dispersion coefficients C_8/C_6 ratio for a set of homonuclear diatomic interactions. Electronic systems data from Jiang et al. (*57*). PsH data taken from Yan et al. (*30*).

3 Quantum Monte Carlo methods

Quantum Monte Carlo (QMC) methods are a family of stochastic techniques used to integrate the many-body time-independent Schrödinger equation over a chosen trial wave function $\Psi_T(\bar{\mathbf{r}})$ (*58–60*), that can also include explicit correlation between the degrees of freedom of the system. Here, with the vector $\bar{\mathbf{r}}$ of $3N_f$ coordinates, we consider the full set of Cartesian coordinates of the system of electrons and positrons.

The simplest of these QMC methods, Variational Monte Carlo (VMC) (*58–60*) is based on the stochastic integration of the energy functional of an Hamiltonian $\hat{H}$ over a variational ansatz $\mathrm{E}\left[\Psi_T\right] = \frac{\int \Psi_T^*(\bar{\mathbf{r}})\hat{\mathrm{H}}\Psi_T(\bar{\mathbf{r}})d\bar{\mathbf{r}}}{\int |\Psi_T(\bar{\mathbf{r}})|^2 d\bar{\mathbf{r}}}$, which is evaluated by separating the integrand $\mathrm{E}\left[\Psi_T\right] = \int E_{\mathrm{loc}}(\bar{\mathbf{r}})\Pi(\bar{\mathbf{r}})d\bar{\mathbf{r}}$, into the product of the probability density $\Pi(\bar{\mathbf{r}}) = \frac{|\Psi_T(\bar{\mathbf{r}})|^2}{\int |\Psi_T(\bar{\mathbf{r}})|^2 d\bar{\mathbf{r}}}$ of finding the system in a configuration $\bar{\mathbf{r}}$ and the local energy $\mathrm{E}_{\mathrm{loc}}(\bar{\mathbf{r}}) = \frac{\hat{\mathrm{H}}\Psi_T(\bar{\mathbf{r}})}{\Psi_T(\bar{\mathbf{r}})}$, that is the energy of the system in that particular configuration.

By computing the local quantities on a sequence of $\mathcal{N}$ uncorrelated configurations, it is possible to obtain the stochastic estimations of the total quantities, such as the energy $\mathrm{E}_{\mathrm{loc}}(\bar{\mathbf{r}})$, through the mean value $\mathrm{E}\left[\Psi_T\right] \approx \langle \mathrm{E}_{\mathrm{loc}} \rangle_{\mathcal{N}} \pm \sqrt{\frac{\left\langle \mathrm{E}_{\mathrm{loc}}^2 \right\rangle_{\mathcal{N}} - \langle \mathrm{E}_{\mathrm{loc}} \rangle_{\mathcal{N}}^2}{\mathcal{N}}}$, with an error that decreases as the $1/\sqrt{\mathcal{N}}$ depending on the variance $\left\langle \mathrm{E}_{\mathrm{loc}}^2 \right\rangle_{\mathcal{N}} - \langle \mathrm{E}_{\mathrm{loc}} \rangle_{\mathcal{N}}^2$.

Within this VMC scheme, we optimize the set of parameters of the wave function, through the Stochastic Reconfiguration procedure described in Refs. 61, 62 with the use of correlated sampling technique (*63*) in order to better estimate the energy variation between parameter updates.

Since, in VMC the accuracy in the description of the ground state is still strongly dependent on the trial wave function $\Psi_T(\bar{\mathbf{r}}; \bar{\alpha})$, and so on the dimension of the variational space (*58–60*). To obtain a more accurate estimation of the physical observable, better describing the dynamical correlation between the particles in the system, it is common to use the DMC method within the Fixed-Node (FN) approximation (*64*) required to overcome the sign problem that arises when dealing with fermionic systems (*65, 66*). The fixed node procedure used in this work follows the standard algorithm in ref. 67 by incorporating several modern features to guarantee better size-consistency properties of the calculations (*68, 69*) and stability (*37*).

In order to evaluate the properties that do not commute with the Hamiltonian, in this work we used the second order estimator (SOE) corrections to reduce the biasses arising from the mixed

averages in FN-DMC (*60, 70*).

4 Mixed-Averages and second order estimations in Diffusion Monte Carlo

In FN-DMC, the time evolution of the product of the trial wave function and the evolved weights generates the mixed distribution $f(\bar{\mathbf{r}}, \tau) = \varphi(\bar{\mathbf{r}}, \tau)\Psi_T(\bar{\mathbf{r}})$, where $\varphi(\bar{\mathbf{r}}, \tau)$ is the best (lower energy) wave function for the ground state with the same nodes as the trial function $\Psi_T(\bar{\mathbf{r}})$. For the energy and operators that commute with the Hamiltonian operator, this 'mixed' estimator corresponds to that obtained on the pure projected state $|\varphi(\bar{\mathbf{r}}, \tau)|^2$, so that

$$E_{\mathrm{MA}} = \frac{\langle \Psi_T|\hat{H}|\varphi\rangle}{\langle \Psi_T|\varphi\rangle} = \frac{\langle \varphi|\hat{H}|\varphi\rangle}{\langle \varphi|\varphi\rangle}, \tag{S1}$$

For all other operators, that do not commute with the Hamiltonian, the mixed average introduces a bias in their estimations. An approximate scheme to reduce this bias, and we show in the work that it is a fundamental correction in all our estimations of the electronic properties, is the second order estimator. This approach assumes that the true ground state is close to the trial wave function so that it can be written as (*60*) a sum of two terms

$$\varphi = \Psi_T + \delta\Psi, \tag{S2}$$

where $\delta\Psi$ is normalized and orthogonal to Ψ_T. In this case the average of a general operator on the projected ground state φ becomes

$$\langle \hat{A}\rangle_{\mathrm{pure}} = \left\langle \varphi|\hat{A}|\varphi\right\rangle = \left\langle \Psi_T|\hat{A}|\Psi_T\right\rangle + 2\left\langle \varphi|\hat{A}|\delta\Psi\right\rangle + \langle \delta\Psi|\hat{A}|\delta\Psi\rangle. \tag{S3}$$

If $\delta\Psi$ is small enough, the second order term $\langle \delta\Psi|\hat{A}|\delta\Psi\rangle$ can be neglected, and after substituting $\left\langle \varphi|\hat{A}|\delta\Psi\right\rangle = \left\langle \Psi_T|\hat{A}|\varphi\right\rangle - \left\langle \Psi_T|\hat{A}|\Psi_T\right\rangle$ we obtain the second order estimator (SOE) as

$$\langle \hat{A}\rangle_{\mathrm{SOE}} \approx 2\langle \hat{A}\rangle_{\mathrm{mix}} - \langle \hat{A}\rangle_{\mathrm{T}}. \tag{S4}$$

Within this approximation, it's essential to construct the most accurate possible VMC wave function. Which is one of our motivation to develop here the extended dynamical Jastrow factor.

5 Hamiltonian of the Electron-positrons systems in weak electrostatic fields

The metastable positron-molecular states, composed of N_n atomic nuclei, N_e electrons (e) and N_p positrons (p), at low energies are described within the Born–Oppenheimer approximation through the fermionic Hamiltonian (in atomic units):

$$\hat{\mathrm{H}}_0 = -\sum_{i=1}^{N_l} \frac{1}{2}\nabla^2_{\mathbf{r}_i} + \sum_{a=1}^{N_n}\sum_{i=1}^{N_l} \frac{Z_a q_i}{|\mathbf{r}_i - \mathbf{R}_a|} + \sum_{j>i=1}^{N_l} \frac{q_i q_j}{|\mathbf{r}_i - \mathbf{r}_j|} + \sum_{b>a=1}^{N_n} \frac{Z_a Z_b}{|\mathbf{R}_a - \mathbf{R}_b|}, \tag{S5}$$

where $N_l = N_e + N_p$ is the total number of leptons, with N_e the number of electrons and N_p the number of positrons, N_n are the number of nuclei in the system, $\{Z_a\}_{a=1}^{N_n}$ are the nuclear charges, $q_i = \pm 1$ are the leptonic charges, and $\bar{\mathbf{r}} = \{\mathbf{r}_i\}_{i=1}^{N_l}$ and $\bar{\mathbf{R}} = \{\mathbf{R}_a\}_{a=1}^{N_n}$ are the coordinate vectors of the leptons and nuclei respectively. In the case of a weak static electric field (*71*), the Hamiltonian of the system

$$\hat{\mathrm{H}} = \hat{\mathrm{H}}_0 - \boldsymbol{\mu} \cdot \mathbf{F} \tag{S6}$$

is written as the sum of the unperturbed Hamiltonian in eq. S5 plus a term that depends on the electrostatic field vector $\mathbf{F}$ and the components of the dipole moment operator $\boldsymbol{\mu}$, which for a system of N_f leptons and N_n nuclei is defined as

$$\boldsymbol{\mu} = \sum_{i=1}^{N_f} q_i \mathbf{r}_i + \sum_{a=1}^{N_n} Z_a \mathbf{R}_a. \tag{S7}$$

6 Electron-positrons Wave function

In the literature, many ansätze have been proposed to describe the many-electrons and many-positrons wave function $\Psi_T(\bar{\mathbf{x}}^p, \bar{\mathbf{x}}^e; \bar{\mathbf{R}})$, where $\bar{\mathbf{x}}^e = \{\bar{\mathbf{r}}^e, \boldsymbol{\sigma}^e\}$ and $\bar{\mathbf{x}}^p = \{\bar{\mathbf{r}}^p, \boldsymbol{\sigma}^p\}$ are the set of Cartesian and spin coordinates for the electrons and positrons respectively, and $\bar{\mathbf{R}}$ is the vector of the nuclear positions. Ideally, the most general expression of the wave function should explicitly include all particle correlation effects, including the nuclei, while establishing the correct spatial and spin symmetries for both the electrons and positrons (*14, 28*).

A first approximation to this explicitly correlated wave function was introduced by Bressanini *et al.* in ref. 72 and was written as a product of pair correlation functions correctly symmetrized or anti-symmetrized to conform with Pauli's exclusion principle. In practice, this ansatz attains high accuracy, but its computational cost rises sharply for systems containing large numbers of atoms and positrons, which can limit its applicability to extensive many-body calculations.

A way to simplify the total wavefunction is to decouple it into the product

$$\Psi(\bar{\mathbf{x}}^e, \bar{\mathbf{x}}^p; \bar{\mathbf{R}}) = \psi_e(\bar{\mathbf{x}}^e; \bar{\mathbf{R}}) \psi_p(\bar{\mathbf{x}}^p; \bar{\mathbf{x}}^e, \bar{\mathbf{R}}) e^{\mathcal{J}(\bar{\mathbf{x}}^e, \bar{\mathbf{x}}^p; \bar{\mathbf{R}})} \tag{S8}$$

of two fermionic functions, one describing the bound states between the electrons and the nuclei $\psi_e(\bar{\mathbf{x}}^e; \bar{\mathbf{R}})$ (such as a Slater determinant) and another one describing the coupling between the electrons and the positrons $\psi_p(\mathbf{x}^p, \bar{\mathbf{x}}^e; \bar{\mathbf{R}})$, and a bosonic Jastrow factor.

One way to further simplify the wavefunction is to assume that the $\psi_p(\bar{\mathbf{x}}^p; \bar{\mathbf{x}}^e, \bar{\mathbf{R}})$ function is independent of $\bar{\mathbf{R}}$ as introduced in ref. 14 and further generalized for QMC in ref. 34, 37.

Following such an approach, in this work $\psi_e(\bar{\mathbf{x}}^e; \bar{\mathbf{R}})$ is built as a Slater determinant, whereas the positronic wave function is described by the electron-positron orbitals originally presented in ref. 34, 37, here now is generalized for multi-positronic systems as

$$\psi_p(\bar{\mathbf{x}}^e, \bar{\mathbf{x}}^p) = \prod_{i=1}^{N_e} \prod_{j=1}^{N_p} \varphi(\mathbf{r}_{ij}^{ep}), \tag{S9}$$

which is based on identical orbitals $\varphi(\mathbf{r}_{ij}^{ep})$ (so that the function is symmetric with respect to the exchange of the electronic coordinates) that depend on the electron-positron distance $\mathbf{r}_i^{ep}$. These orbitals φ are built as linear combinations

$$\varphi(\mathbf{r}^{ep}) = \sum_{q=1}^{Q} l_q \phi_q \left(\mathbf{r}^{ep}\right) \tag{S10}$$

of electron-positron orbitals

$$\phi\left(\mathbf{x}^{ep}\right) = r^{ep} R\left(r^{ep}\right) Y_l^m(\theta^{ep}, \phi^{ep}). \tag{S11}$$

Finally, the Jastrow factor consists of the two-body terms between fermions and fermion-nuclei to describe their cusp conditions and pair correlations, together with the full three- and four-body dynamical Jastrow to account for homogeneous and non-homogeneous two-body correlations in the field of the nuclei or positrons as

$$\mathcal{J}(\bar{\mathbf{x}}^e, \bar{\mathbf{x}}^p; \bar{\mathbf{R}}) = \mathcal{J}_c(\bar{\mathbf{x}}^e, \bar{\mathbf{x}}^p; \bar{\mathbf{R}}) + \mathcal{J}_{3/4}(\bar{\mathbf{r}}^e, \bar{\mathbf{r}}^p; \bar{\mathbf{R}}). \tag{S12}$$

Since the $\mathcal{J}_c(\bar{\mathbf{x}}^e, \bar{\mathbf{x}}^p, \bar{\mathbf{R}})$ term is extensively discussed in refs. 27, 34, 37 we will not recall its basic form, on the other hand we will concentrate on the dynamical Jastrow factor $\mathcal{J}_d(\bar{\mathbf{r}}^e, \bar{\mathbf{r}}^p; \bar{\mathbf{R}})$ and its extension.

7 Three-Four body Jastrow factor for electron-positron correlations

7.1 Three-/Four- body Jastrow factor

A first version of the dynamical Jastrow factor for electron-positron systems was introduced in ref. 34, inspired by the purely electronic one of Casula *et al.* in ref. 73, as a linear combination of products of atomic orbitals centered on the nuclei. Although this is not the most common expression of the three-/four-body correlation term (*74, 75*), the advantages of this formulation lie in its simplicity and computational efficiency (*73*). Yet, this Jastrow explicitly depends only on the distances between the nuclei and the fermions. While this is a reasonable correlation factor to describe the attractiveness between nuclei and electrons, it becomes less efficient for the positrons that are actually attracted by the electrons.

In order to further generalize this Jastrow, we thus couple the atomic orbitals with a set of positronic orbitals that explicitly depend on the relative distances between electrons and positrons. Therefore, this new dynamical Jastrow factor is built as a combination of three groups of terms

$$\mathcal{J}_d(\bar{\mathbf{r}}^e, \bar{\mathbf{r}}^p; \bar{\mathbf{R}}) = \mathcal{A}(\bar{\mathbf{r}}^e, \bar{\mathbf{r}}^p; \bar{\mathbf{R}}) + \mathcal{G}(\bar{\mathbf{r}}^e, \bar{\mathbf{r}}^p) + \mathcal{M}(\bar{\mathbf{r}}^e, \bar{\mathbf{r}}^p; \bar{\mathbf{R}}). \tag{S13}$$

which correspond to the atomic Jastrow $\mathcal{A}(\bar{\mathbf{r}}^e, \bar{\mathbf{r}}^p; \bar{\mathbf{R}})$, built purely from the combination of atomic orbitals as previously used in refs. 27, 34, a set of terms $\mathcal{G}(\bar{\mathbf{r}}^e, \bar{\mathbf{r}}^p)$ constructed only from the positronic orbitals, and a mixed term that combines both orbital sets, $\mathcal{M}(\bar{\mathbf{r}}^e, \bar{\mathbf{r}}^p; \bar{\mathbf{R}})$.

A schematic representation of all the terms in the Jastrow can be found in Fig. S4. In the section that follows, we will focus on the justifications that have led to the choice of constructing the three-/four- body Jastrow with all these set of terms.

7.2 Atomic dynamical Jastrow Factor

The first part of the dynamical Jastrow, already introduced for multi-positronic systems in ref. 34, is built on a basis set of Q non-normalized atomic orbitals $\chi_\nu(\mathbf{r})$, that are centered on different atoms (the atomic index is included in the orbital index μ or ν and will always be omitted in the following sections).

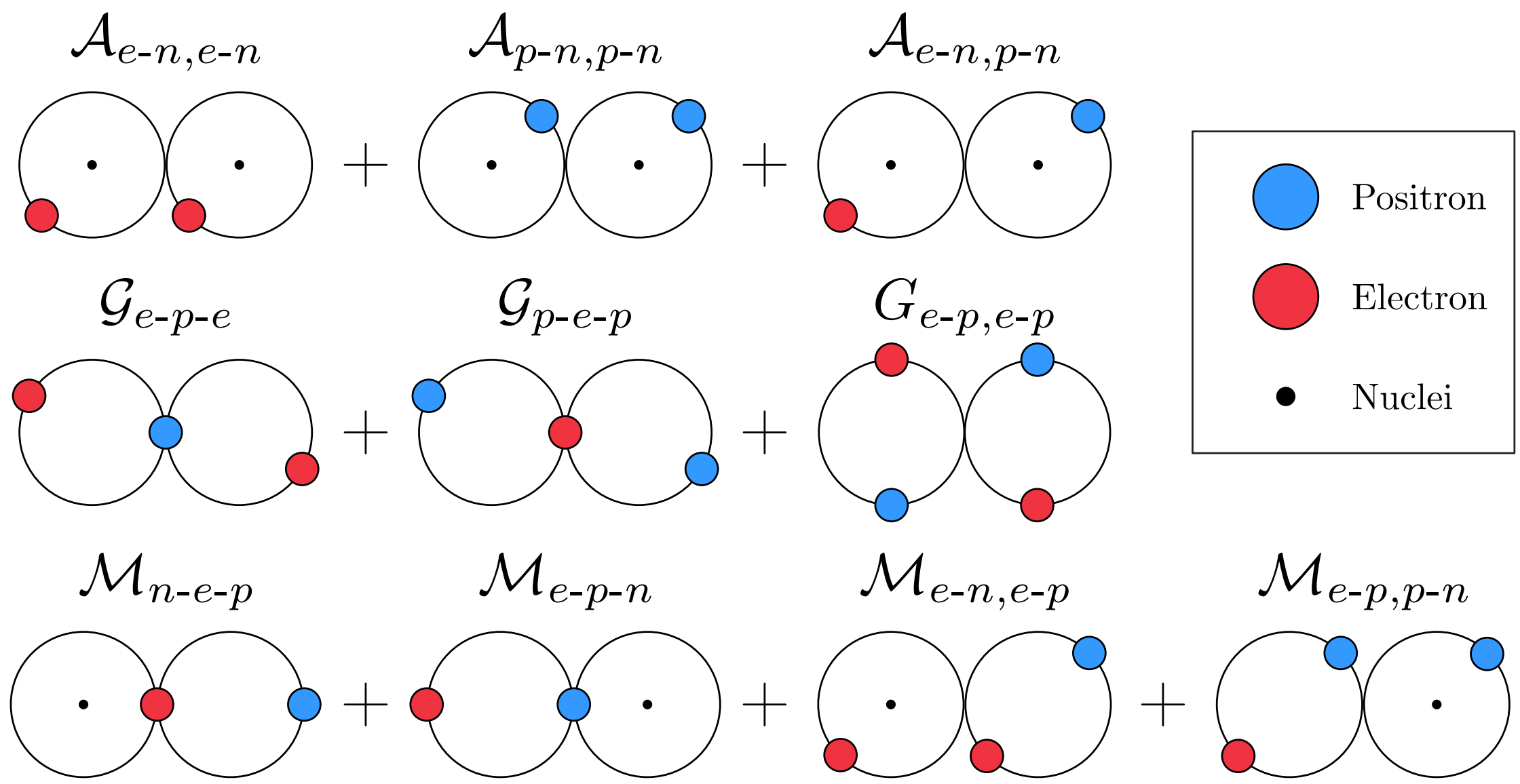

Figure S4: **Schematic representation of the dynamical Jastrow factor.**
$\mathcal{A}$ terms correspond to the Atomic dynamical Jastrow, $\mathcal{G}$ terms for Germinal Electron-Positron dynamical Jastrow, and finally the $\mathcal{M}$ terms for the Mixed Atomic and Geminal dynamical Jastrow. The *hyphen* mark is used to represent the distance between two particles within an orbital, and the *comma* symbol to indicate a product of two uncoupled orbitals.

For a system comprised of N_e electrons and N_p positrons, this part of the Jastrow factor consists of the sum of three groups of correlation functions,

$$\mathcal{A}(\bar{\mathbf{r}}^e, \bar{\mathbf{r}}^p; \bar{\mathbf{R}}) = \sum_{j>i}^{N_e} \mathcal{A}^{ee}(\mathbf{r}_i^e, \mathbf{r}_j^e; \bar{\mathbf{R}}) + \sum_{h>t}^{N_p} \mathcal{A}^{pp}(\mathbf{r}_h^p, \mathbf{r}_t^p; \bar{\mathbf{R}}) + \sum_{i}^{N_e} \sum_{h}^{N_p} \mathcal{A}^{ep}(\mathbf{r}_i^e, \mathbf{r}_h^p; \bar{\mathbf{R}}), \tag{S14}$$

all written as linear combination of products of two atomic orbitals. The first set will correlate two electrons

$$\mathcal{A}^{ee}(\mathbf{r}_i^e, \mathbf{r}_j^e; \bar{\mathbf{R}}) = \sum_{\mu,\nu}^{Q} A_{\mu\nu}^{ee} \chi_\mu(\mathbf{r}_i^e) \chi_\nu(\mathbf{r}_j^e) \tag{S15}$$

the second set will correlate two positrons

$$\mathcal{A}^{pp}(\mathbf{r}_h^p, \mathbf{r}_t^p; \bar{\mathbf{R}}) = \sum_{\mu,\nu}^{Q} A_{\mu\nu}^{pp} \chi_\mu(\mathbf{r}_h^p) \chi_\nu(\mathbf{r}_t^p), \tag{S16}$$

and the third set will correlate one electron and one positron

$$\mathcal{A}^{ep}(\mathbf{r}_i^e, \mathbf{r}_h^p; \bar{\mathbf{R}}) = \sum_{\mu,\nu}^{Q} A_{\mu\nu}^{ep} \chi_\mu(\mathbf{r}_i^e) \chi_\nu(\mathbf{r}_h^p). \tag{S17}$$

Here the square matrices $\mathbf{A}^{ee}$, $\mathbf{A}^{pp}$, and $\mathbf{A}^{ep}$ that contain the linear coefficients of the expansions, $A^{ee}_{\mu\nu}$, $A^{pp}_{\mu\nu}$, and $A^{ep}_{\mu\nu}$ respectively, describe the coupling between the various atomic orbitals occupied by different fermionic pairs. To avoid spin contamination, the $\mathbf{A}^{ee}$ and $\mathbf{A}^{pp}$ matrices must be symmetric, *ie* $A^{ee}_{\mu\nu} = A^{ee}_{\nu\mu}$ and $A^{pp}_{\mu\nu} = A^{pp}_{\nu\mu}$, in order for the Jastrow to be invariant with respect to the exchange of two electrons or two positrons, no matter their spin.

Despite the quality of the results presented in refs. 27, 34 these Jastrow terms only contain functions of the electron-nuclei and positron-nuclei distances with no explicit correlation between the two attractive fermionic particles (electrons and positrons).

Yet, the correlation effects arising from explicit electron-positron terms are crucial, especially when describing homogeneous systems of pure electron-positron pairs, or for systems with large numbers of electrons where the positron particle is not in general spherically distributed around an atom (*20, 25, 27, 28, 34, 76*).

For this reason, in the following section we introduce additional components based on electron-positron (or positronic) orbitals (*34, 77*).

7.3 Geminal dynamical Jastrow Factor

As previously discussed, the dynamical Jastrow factor constructed as a linear combination of atomic orbitals does not explicitly include the correlation between electron-positron pairs. These pair correlation functions are essential, for example, when describing homogeneous systems of pure electrons and positrons, such as the positronium anion (Ps^-) or the positronium dimer (Ps_2), or in those cases in which, for large molecules, the positrons' wave function is not spherically symmetric around the nuclei.

For this reason, we now introduce additional terms that can be indicated as the Geminal Jastrow factor, which are built through a set of K non-normalized positronic orbitals (*34*)

$$\varphi_\eta\left(\mathbf{r}^{ep}_{ih}\right) = r^{ep}_{ih} R\left(r^{ep}_{ih}\right) Y^m_l(\theta^{ep}_{ih}, \phi^{ep}_{ih}), \tag{S18}$$

that depend on the distance vector $\mathbf{r}^{ep}_{ih} = \mathbf{r}^e_i - \mathbf{r}^p_h$ connecting the electron-positron pair, and where $Y^m_l(\theta^{ep}_{ih}, \phi^{ep}_{ih})$ are cubic harmonics, and $R\left(r^{ep}_{ih}\right)$ are non-normalized Gaussian type orbitals. For convention, we chose these positronic orbitals to be centered on the positron's position.

As for the atomic Jastrow, we now describe the three-/four- body correlations between two pairs of electron-positron couples as the sum of three sets of correlation functions

$$\mathcal{G}(\bar{\mathbf{r}}^e,\bar{\mathbf{r}}^p) = \sum_{j>i}^{N_e}\sum_{h}^{N_p}\mathcal{G}^{epe}(\mathbf{r}_i^e,\mathbf{r}_h^p,\mathbf{r}_j^e) + \sum_{t>h}^{N_p}\sum_{i}^{N_e}\mathcal{G}^{pep}(\mathbf{r}_h^p,\mathbf{r}_i^e,\mathbf{r}_t^p) + \sum_{j>i}^{N_e}\sum_{t>h}^{N_p}\mathcal{G}^{epep}(\mathbf{r}_i^e,\mathbf{r}_h^p,\mathbf{r}_j^e,\mathbf{r}_t^p), \tag{S19}$$

each written as the linear combination of products of two positronic orbitals. Here, the first

$$\mathcal{G}^{epe}(\mathbf{r}_i^e,\mathbf{r}_h^p,\mathbf{r}_j^e) = \sum_{\eta,\tau}^{K} G_{\eta\tau}^{epe}\varphi_\eta(\mathbf{r}_{ih}^{ep})\varphi_\tau(\mathbf{r}_{jh}^{ep}) \tag{S20}$$

and second

$$\mathcal{G}^{pep}(\mathbf{r}_h^p,\mathbf{r}_i^e,\mathbf{r}_t^p) = \sum_{\eta,\tau}^{K} G_{\eta\tau}^{pep}\varphi_\eta(\mathbf{r}_{ih}^{ep})\varphi_\tau(\mathbf{r}_{it}^{ep}) \tag{S21}$$

set of terms represents three fermion correlations respectively between two electrons and one positron (eq. S20) and two positrons and one electron (eq. S21).

Since the Jastrow must be invariant with respect to the exchange of two electronic or positronic coordinates, the square matrices of the coefficients that define the two expansions, $\mathbf{G}^{epe}$ and $\mathbf{G}^{pep}$, must symmetric, *ie* $G_{\eta\tau}^{epe} = G_{\tau\eta}^{epe}$ and $G_{\eta\tau}^{pep} = G_{\tau\eta}^{pep}$.

The third set of terms describes the correlation between four different fermions: two electrons and two positrons, again as a product of two geminal orbitals,

$$\mathcal{G}^{epep}(\mathbf{r}_i^e,\mathbf{r}_h^p,\mathbf{r}_j^e,\mathbf{r}_t^p) = \sum_{\eta,\tau}^{K} G_{\eta\tau}^{epep}\left[\varphi_\eta(\mathbf{r}_{ih}^{ep})\varphi_\tau(\mathbf{r}_{jt}^{ep}) + + \varphi_\eta(\mathbf{r}_{jh}^{ep})\varphi_\tau(\mathbf{r}_{it}^{ep})\right]. \tag{S22}$$

As for the previous two terms, the four-particle correlation function must be symmetric with respect to the exchange of both electrons and positrons, and in order to impose this not only the coupling matrix $\mathbf{G}^{epep}$ must be symmetric, *ie* $G_{\eta\tau}^{epep} = G_{\tau\eta}^{epep}$, but also we need to symmetrize the terms with respect to the exchange of both electron pairs and positron pairs, and for this reason we must write them as the sum of the two contributions $\varphi_\eta(\mathbf{r}_{ih}^{ep})\varphi_\tau(\mathbf{r}_{jt}^{ep}) + \varphi_\eta(\mathbf{r}_{jh}^{ep})\varphi_\tau(\mathbf{r}_{it}^{ep})$.

7.4 Mixed atomic-geminal dynamical Jastrow Factor

The atomic and geminal Jastrow factors defined in the previous sections do not exhaust the set of four-body correlations that we can describe with the atomic and positronic basis sets. While the atomic part neglects the pair correlation between electron-positron pairs, and the Geminal part

neglects the influence of the nuclei, we can construct a third term that describes the correlation of two or three fermions in the field of one nucleus, as products of positronic and atomic orbitals.

First, we define the coupling of the ith electron with the hth positron in the field of the nuclear positions. This correlation is constructed as the sum of two combinations, the first of which is the term

$$\mathcal{M}^{nep}(\mathbf{r}_i^e, \mathbf{r}_h^p; \bar{\mathbf{R}}) = \sum_{\nu}^{Q} \sum_{\eta}^{K} M_{\nu\eta}^{nep} \chi_\nu(\mathbf{r}_i^e) \varphi_\eta(\mathbf{r}_{ih}^{ep}) \tag{S23}$$

that correlates the ith electron with the nuclear coordinates through the atomic basis set, and with the hth positron through the positronic basis set. The second term inverts the couplings, correlating the positron with the atomic basis and with the electron through the positronic basis set:

$$\mathcal{M}^{epn}(\mathbf{r}_i^e, \mathbf{r}_h^p; \bar{\mathbf{R}}) = \sum_{\nu}^{Q} \sum_{\eta}^{K} M_{\nu\eta}^{epn} \varphi_\eta(\mathbf{r}_{ih}^{ep}) \chi_\nu(\mathbf{r}_h^p). \tag{S24}$$

Following the same procedure we can build terms correlating three fermions in the field of the nuclei. The first set of terms will correlate two electrons and a positron in the field of the nuclei,

$$\mathcal{M}^{enep}(\mathbf{r}_i^e, \mathbf{r}_j^e, \mathbf{r}_h^p; \bar{\mathbf{R}}) = \sum_{\nu}^{Q} \sum_{\eta}^{K} M_{\nu\eta}^{enep} \chi_\nu(\mathbf{r}_i^e) \varphi_\eta(\mathbf{r}_{jh}^{ep}), \tag{S25}$$

while the second set of terms will correlate two positrons and one electron:

$$\mathcal{M}^{eppn}(\mathbf{r}_i^e, \mathbf{r}_h^p, \mathbf{r}_t^p; \bar{\mathbf{R}}) = \sum_{\nu}^{Q} \sum_{\eta}^{K} M_{\nu\eta}^{eppn} \varphi_\eta(\mathbf{r}_{ih}^{ep}) \chi_\nu(\mathbf{r}_t^p). \tag{S26}$$

Since the first two sets of terms are two-body correlation functions coupling an electron and a positron no symmetries have to be imposed in the rectangular matrices, $\mathbf{M}^{nep}$ and $\mathbf{M}^{epn}$. The same hold for the rectangular matrices $\mathbf{M}^{enep}$ and $\mathbf{M}^{eppn}$ that appear in the last two three-body terms. Yet, here we must guarantee the invariance of the Jastrow factor for the exchange between the two electrons in eq. S25 and the two positrons in eq. S26. In order to do so, it is sufficient to extend the sums to all pairs of different fermions. Therefore, using eqs. S24, S23, S25, and S26 the total mixed atomic-geminal dynamical factor is expressed as:

$$\begin{aligned}\mathcal{M}(\bar{\mathbf{r}}^e, \bar{\mathbf{r}}^p; \bar{\mathbf{R}}) = &\sum_{i}^{N_e} \sum_{h}^{N_p} \mathcal{M}^{nep}(\mathbf{r}_i^e, \mathbf{r}_h^p; \bar{\mathbf{R}}) + \sum_{i}^{N_e} \sum_{h}^{N_p} \mathcal{M}^{epn}(\mathbf{r}_i^e, \mathbf{r}_h^p; \bar{\mathbf{R}}) + \sum_{j\neq i}^{N_e} \sum_{h}^{N_p} \mathcal{M}^{enep}(\mathbf{r}_i^e, \mathbf{r}_j^e, \mathbf{r}_h^p; \bar{\mathbf{R}}) \\ &+ \sum_{i}^{N_e} \sum_{t\neq h}^{N_p} \mathcal{M}^{eppn}(\mathbf{r}_i^e, \mathbf{r}_h^p, \mathbf{r}_t^p; \bar{\mathbf{R}}).\end{aligned} \tag{S27}$$

In Table S1, we reported the convergence of the VMC energy for PsH with respect to the size of the atomic and geminal Jastrow basis. Those results help us to establish an adequate compromise between correlation energy gained and computational cost.

Table S1: **Jastrow basis set convergence.**

Effect of the size of the atomic Jastrow basis and Geminal electron-positron basis on the VMC energy for PsH (in Hartree units) using the full dynamical Jastrow (atomic, geminal and mixed) see main text. The ΔE column corresponds to the energy difference between each basis set combination and the highest energy obtained.

$\mathcal{A}$ **Basis**	$\mathcal{G}$ **Basis**	**VMC [Ha]**	**ΔE [mHa]**
1s	1s	-0.784278(19)	0.0
1s	3s	-0.784856(19)	-0.6
1s	3s2p	-0.784797(18)	-0.5
1s	3s2p1d	-0.784794(19)	-0.5
3s	1s	-0.785832(16)	-1.6
3s	3s	-0.786660(19)	-2.4
3s	3s2p	-0.786674(12)	-2.4
3s	3s2p1d	-0.786675(15)	-2.4
3s2p	1s	-0.787104(14)	-2.8
3s2p	3s	-0.787620(15)	-3.3
3s2p	3s2p	-0.788107(12)	-3.8
3s2p	3s2p1d	-0.788111(10)	-3.8
3s2p1d	1s	-0.787131(14)	-2.9
3s2p1d	3s	-0.787630(16)	-3.4
3s2p1d	3s2p	-0.788159(9)	-3.9
3s2p1d	3s2p1d	-0.788203(13)	-3.9

8 External electric field and response properties

8.1 External electric field

In the QMC formalism, the local dipole moment corresponds simply to:

$$\mu_{a,loc}(\bar{\mathbf{r}}) = \frac{\hat{\mu}\Psi_T(\bar{\lambda};\bar{\mathbf{r}})}{\Psi_T(\bar{\lambda};\bar{\mathbf{r}})} = \sum_{i}^{N_p} q_i r_{i,a}. \tag{S28}$$

Therefore, the local energy corresponding to the Hamiltonian of a system in the presence of a static uniform external electric field can be expressed as

$$E^F_{loc}(\bar{\lambda};\bar{\mathbf{r}}) = E^0_{loc}(\bar{\lambda};\bar{\mathbf{r}}) - \sum_a \mu_{a,loc}(\bar{\mathbf{r}}) F_a \tag{S29}$$

Here, it should be noted that the contributions from the external field do not depend on the set of variational parameters $\overline{\lambda}$ of the trial wave function $\Psi_T(\bar{\lambda};\bar{\mathbf{r}})$.

8.2 Polarizabilities

Considering that the energy of a system under a weak external electric field can be expanded in a power series with respect to the strength of the electric field (*71*) , in this work we computationally estimated the z-th component, that for the PsH dimer corresponds to the component along the molecular bond, of the dipole polarizabilities by performing a 4th- or 3th-degree polynomial interpolation of the total energies or dipole moment, for a set of different strengths of an external electric field $\vec{F} = \{0, 0, F_z\}$ varying from 0.001 to 0.007 Hartree·e^{-1}·bohr^{-1}, according to

$$E(F_z) = E_0 - \mu_z^{(0)} F_z - \frac{1}{2!}\alpha_{zz} F_z^2 - \frac{1}{3!}\beta_{zzz} F_z^3 - \frac{1}{4!}\gamma_{zzzz} F_z^4 + \ldots, \tag{S30}$$

where γ, β, α, $\mu_z^{(0)}$, and E_0 are the fitting parameters. Therefore, the second derivative at $\vec{F} = 0$ corresponds to the dipole polarizability,

$$\alpha_{ab} = -\left(\frac{\partial^2 E(F_{ab})}{\partial F_{ab}^2}\right)_{F_{ab}=0}. \tag{S31}$$

Analogously, the dipole moment can be expanded as

$$\mu_z(\vec{F}) = -\frac{1}{3!}\gamma_{zzzz} F_z^3 - \frac{1}{2!}\beta_{zzz} F_z^2 - \alpha_{zz} F_z - \mu_z^{(0)} \tag{S32}$$

where the first derivative at $\vec{F} = 0$ corresponds to the dipole polarizability.

$$\alpha_{ab} = \left(\frac{\partial \mu_b(F_a)}{\partial F_a} \right)_{F_a=0} . \tag{S33}$$

For $(PsH)_2$, the only non-spherical symmetric system, the hydrogen nuclei were aligned along the z-axis, therefore, the α_{zz} component corresponds to its parallel component $\alpha_{\parallel}$.

Here we performed a systematic analysis on different computation schemes for the dipole polarizability. By analyzing the convergence and estimated error with respect to reference values (See figure S5), we found that the best performing approach is the 4th-degree polynomial fitting of the energies or 3rd-degree of the dipole, employing the higher interval of electric field strength of 0.007 a.u.

On the other hand, a 2nd-degree polynomial fitting of the energies or 1st- degree of the dipole can also converge to reference values at smaller strengths of the field, but with higher estimated error bars due to the small energy or dipole differences compared to their QMC stochastic error.

Remarkably, all computed polarizabilites are in agreement within their error bars for all the tested DMC time-steps between 0.001 and 0.015 a.u. (See figure S2). This fast convergence suggests that future polarizability studies with QMC could employ the less expensive calculations with higher time steps.

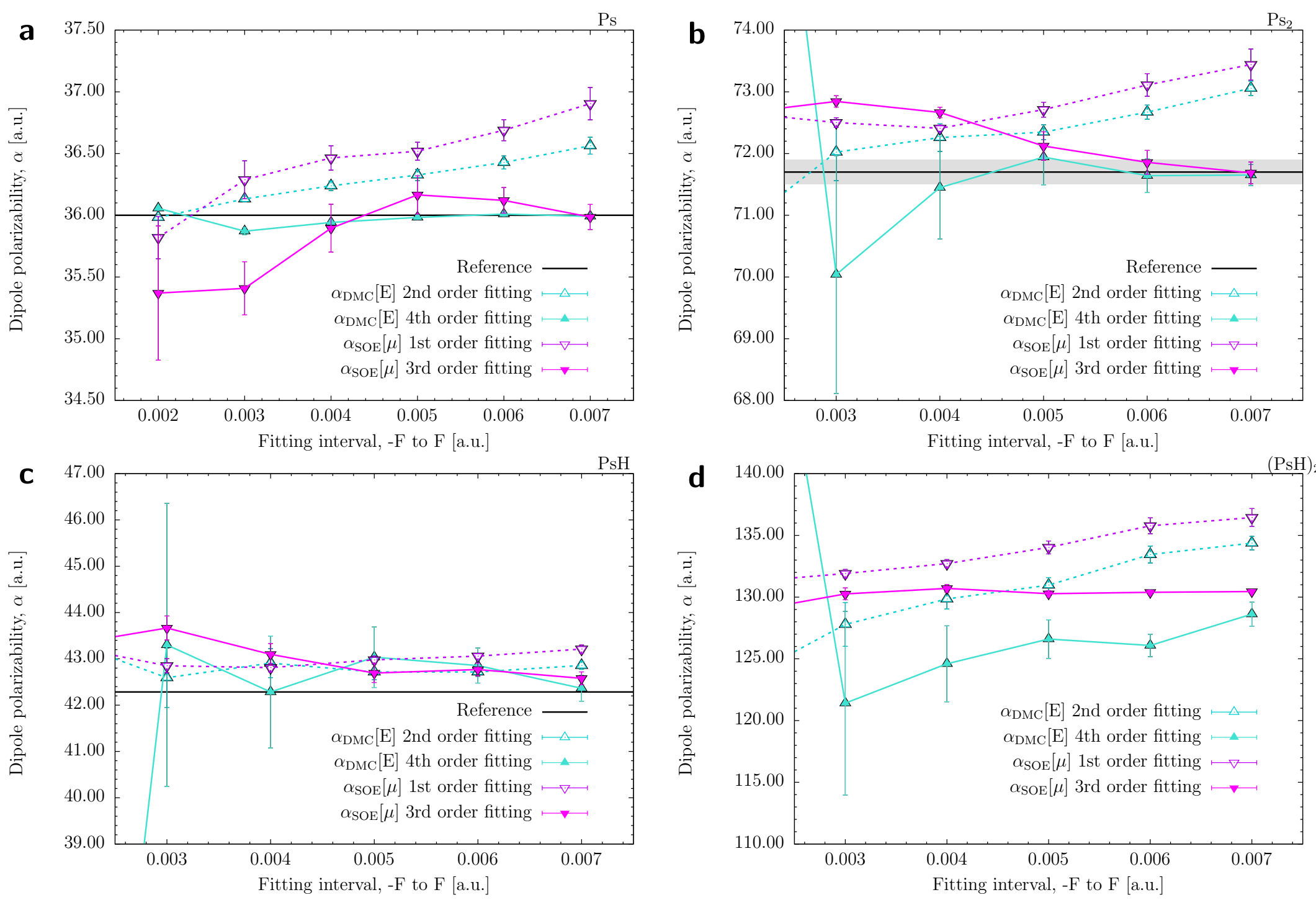

Figure S5: **Polarizability convergence with respect to electric field strength,** F**.**

Dipole polarizabilities for Ps (**a**), Ps_2 (**b**), PsH (**c**), $(PsH)_2$ (**d**) obtained from fitting of 2nd- and 4th-degree polynomial of the energy, as well as from fitting of 1st- and 3rd-degree polynomial of the total dipole as a function of the strength electric field F, for a set of different intervals $-F$ to F spaced every 0.001 a.u. The DMC values computed with a time-step of 0.001 a.u. Ps2 reference polarizability from Hylleraas wave function calculations of Ref. 30, and PsH polarizability from PIMC of Ref. 78.

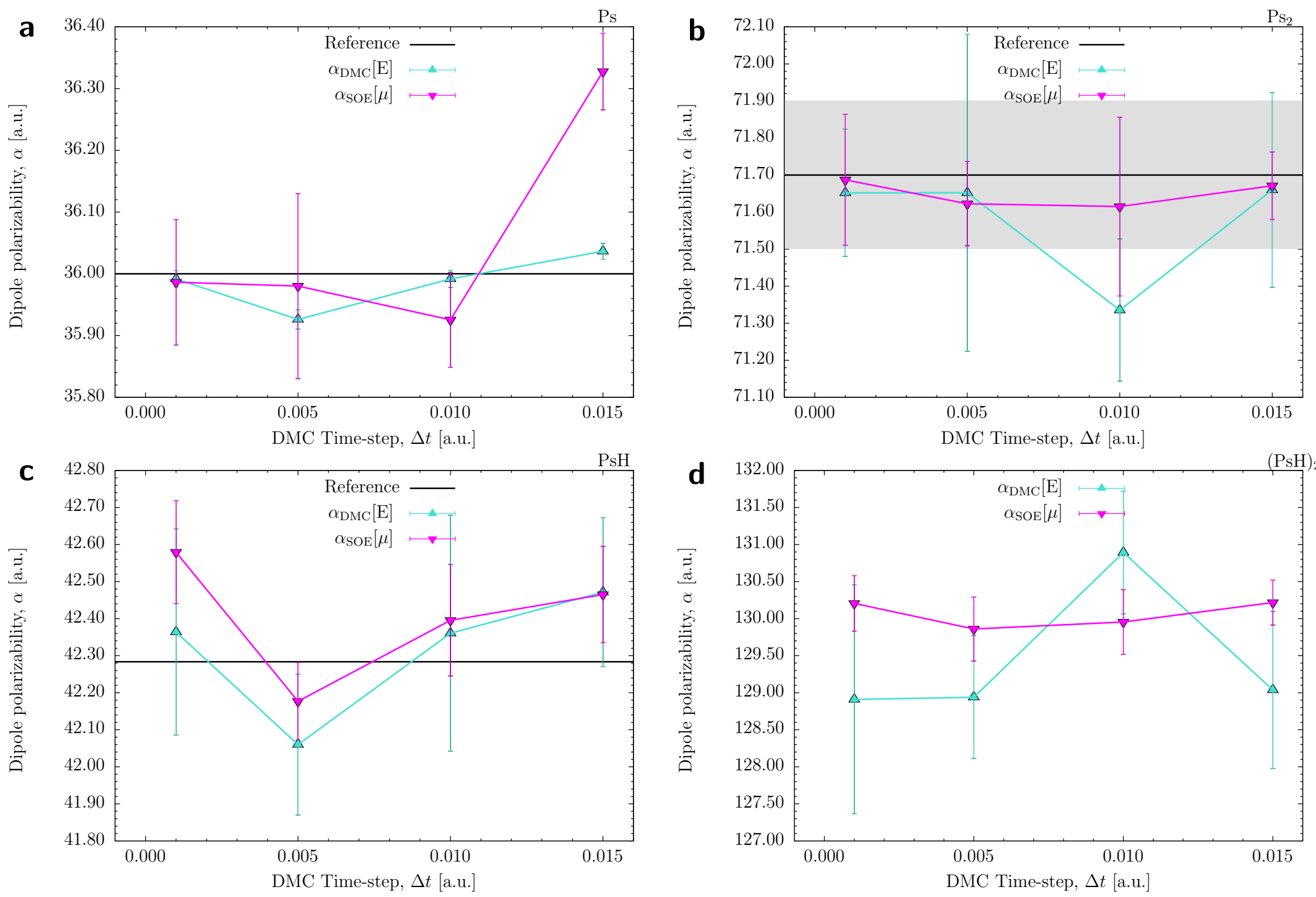

Figure S6: **Polarizability convergence with respect to the DMC time-step Δt.**

Dipole polarizabilities for Ps (**a**), Ps_2 (**b**), PsH (**c**), $(PsH)_2$ (**d**) obtained from fitting of 4th-degree polynomial of the energy, as well as from fitting of 3rd-degree polynomial of the total dipole as a function of the strength electric field F between -0.007 to 0.007 a.u. Ps2 reference polarizability from Hylleraas wave function calculations of Ref. 30, and PsH polarizability from PIMC of Ref. 78.

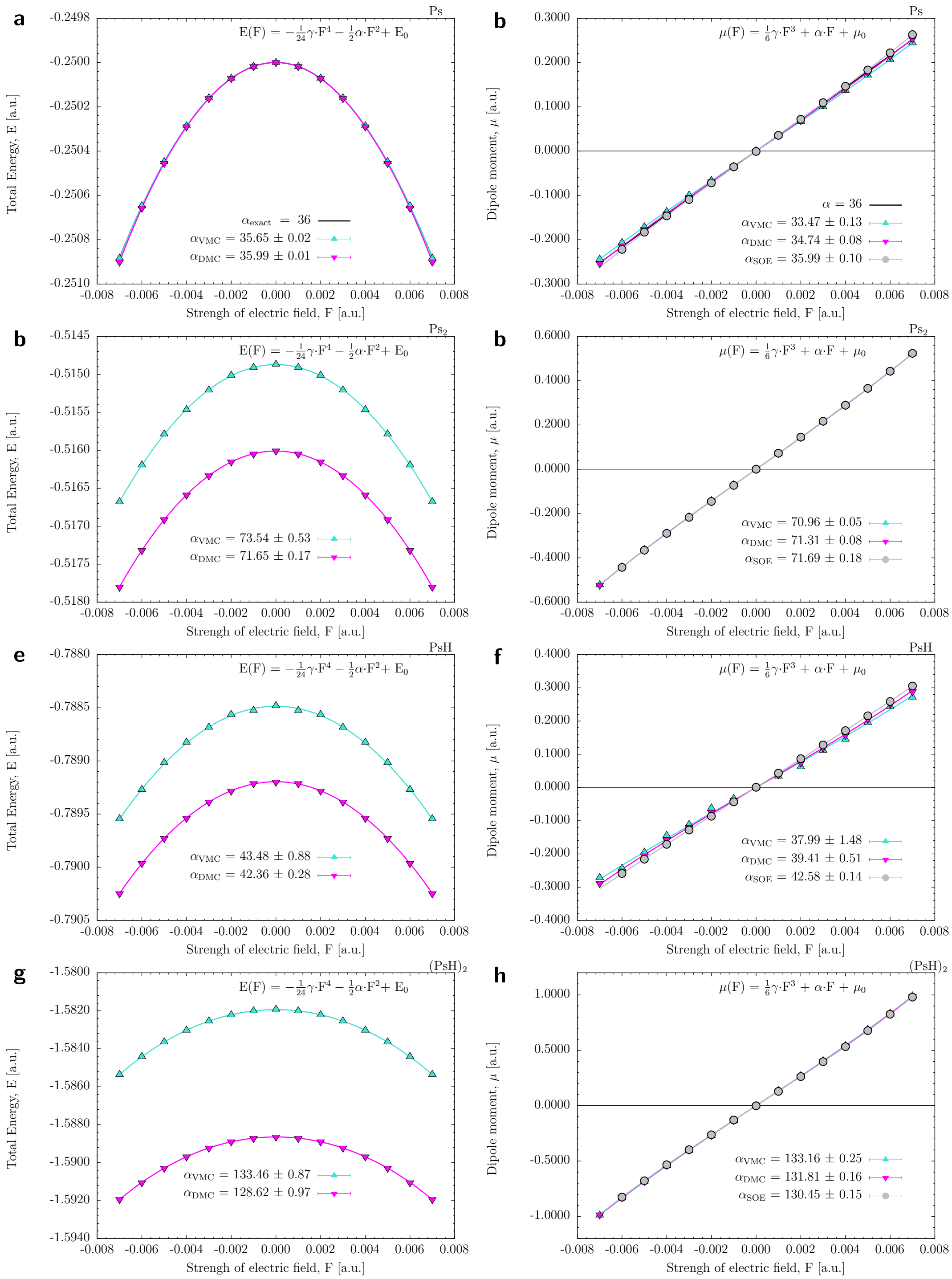

Figure S7: **Electric strength effect on energies and dipole moment.**

Total energies (first column) and dipole moment (second column) as a function of the strength of an external electric field for **a-b**: Ps, **c-d**: Ps_2, **e-f**: PsH and **g-h**: $(PsH)_2$ (parallel component) computed at VMC (cyan), DMC (magenta), and mixed estimator, MXD (gray). Solid lines indicate the quadratic or linear fit. All error bars are included.

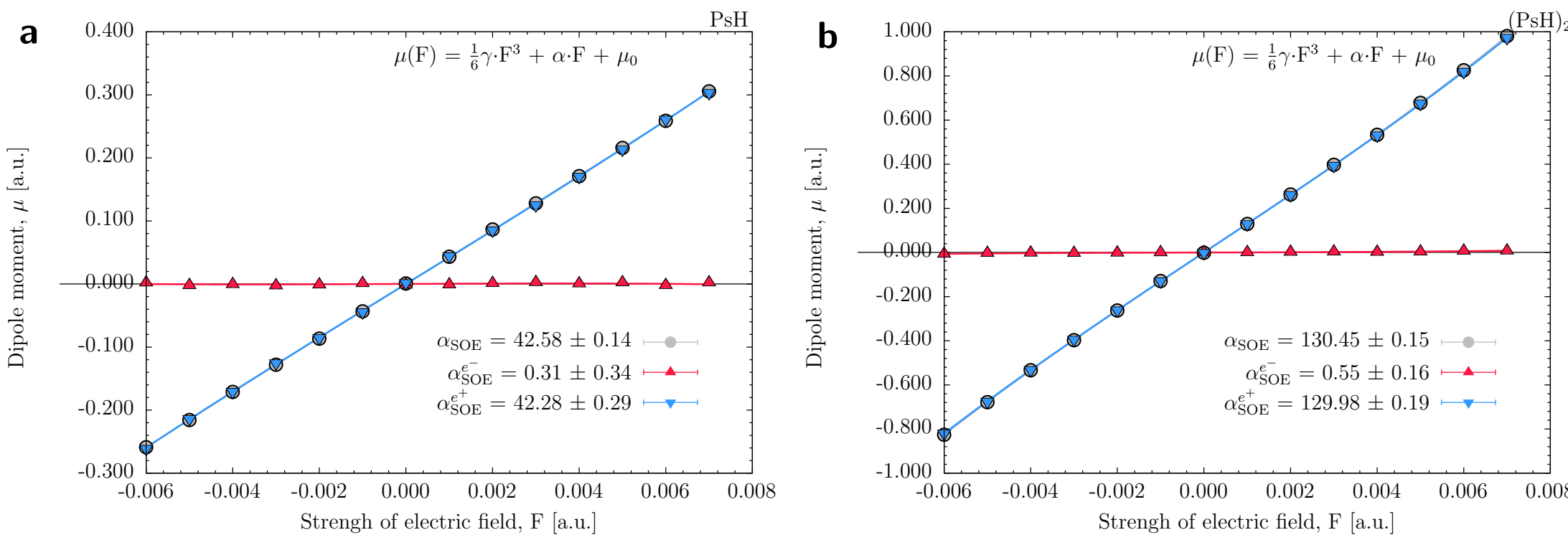

Figure S8: **Electronic and positronic dipole polarizability contribution.**

Decomposition of the dipole moment (grey) into electronic (red) and positronic (blue) displacement from the nuclei as a function of the strength of an external electric field for **a**: PsH and **b**: $(PsH)_2$ (parallel component).

9 Annihilation rate

For a closed-shell electronic and positronic system, the dominant annihilation path is the two-photon process. Thus, the rate at which an electron and a positron in a singlet state come into direct contact is given by the two-photon annihilation rate (*79*)

$$\Gamma_2 = \pi r_0^2 c \left\langle \delta_{ep} \right\rangle , \tag{S34}$$

which is proportional to the electron-positron contact density, related to the probability of finding the electron and the positron at a certain point in space

$$\left\langle \delta_{ep} \right\rangle = \left\langle \Psi \left(\mathbf{r}_p, \mathbf{r}_e \right) \middle| \sum_{i=1}^{N_e} \sum_{j=1}^{N_p} \delta \left(\mathbf{r}_i - \mathbf{r}_j \right) \middle| \Psi \left(\mathbf{r}_p, \mathbf{r}_e \right) \right\rangle . \tag{S35}$$

here c is the speed of light in vacuum and r_0 is the classical electron radius, which is connected to the Bohr radius a_0 by $r_0 = \alpha_{fsc}^2 a_0$, where α_{fsc} is the fine structure constant.

In QMC, it is extremely expensive to properly sample the coalescence integral in the annihilation rate of equation S35 because of the low number of configurations for which an electron and a positron are located at the same point, or close to it. For that reason, it is preferred to use the simple extrapolation technique proposed by Jiang *et al.* in Ref. (*80*), where the coalescence $\delta \left(\mathbf{r}_i - \mathbf{r}_j \right)$ is replaced by

$$\delta_a \left(x_i - x_j \right) = \begin{cases} \frac{3}{4\pi a^3}, & \text{for } \left| x_i - x_j \right| \leqslant a \\ 0, & \text{otherwise} \end{cases} . \tag{S36}$$

Therefore, the quantity $\Gamma_2(a)$ should be computed and stored for a set of different distance values a by summing the contribution of each walkers with an electron-positron distance lower than a. Afterwards, the QMC averages of $\Gamma_2(a)$ are extrapolated to $a = 0$ by doing a standard linear fit considering the standard error of the averages. However, it should be noted that when a is small, the deviation of the counts becomes larger because the counts are few.

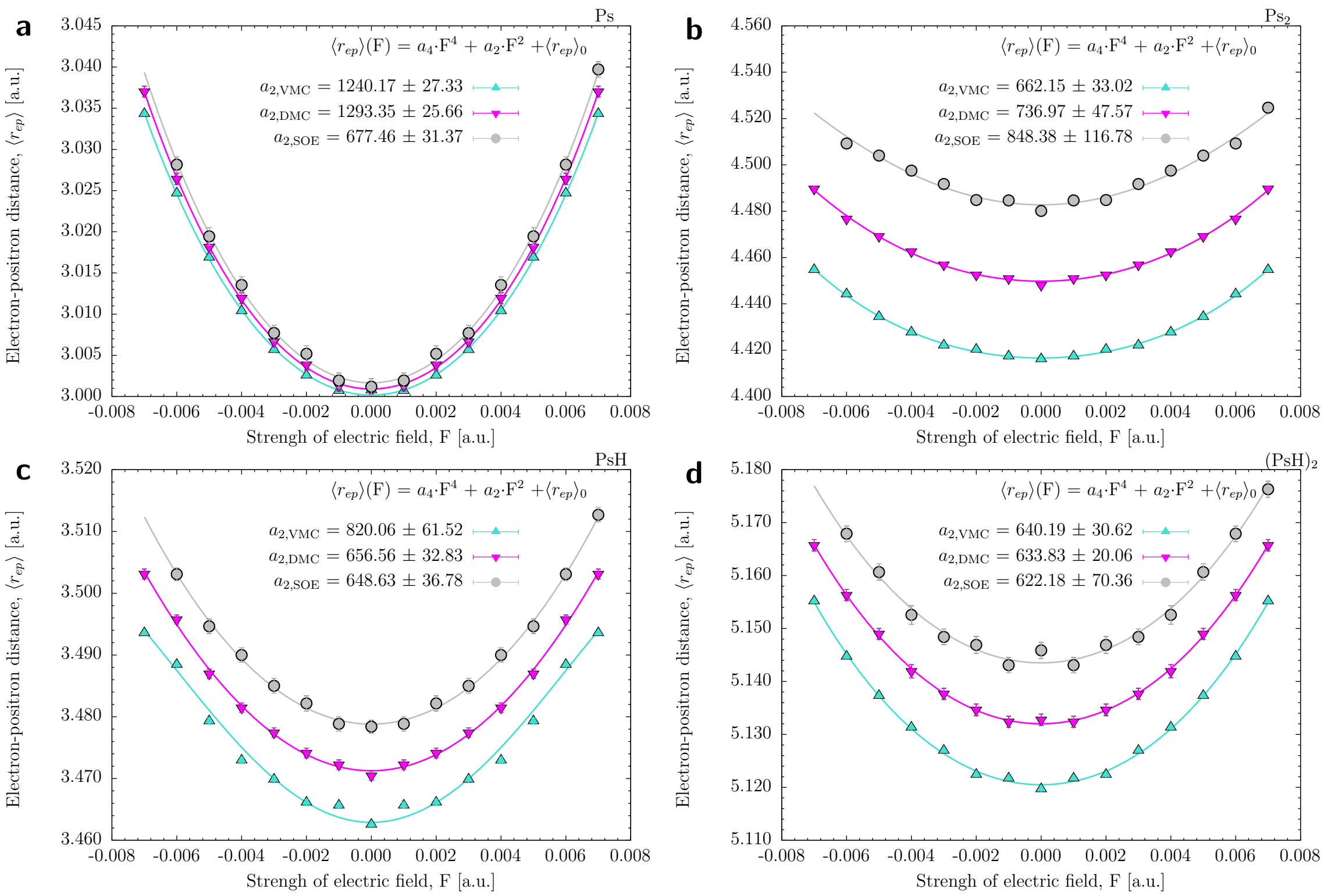

Figure S9: **Electron-positron distance response.**

Polynomial fitting of the expectation values of the electron-positron distance as a function of the strength of an external electric field, computed with VMC, DMC, and SOE for positronium: Ps (**a**), positronium dimer: Ps_2 (**b**), positronium hydride: PsH (**c**), and positronium hydride dimer: $(PsH)_2$ in the parallel component (**d**).

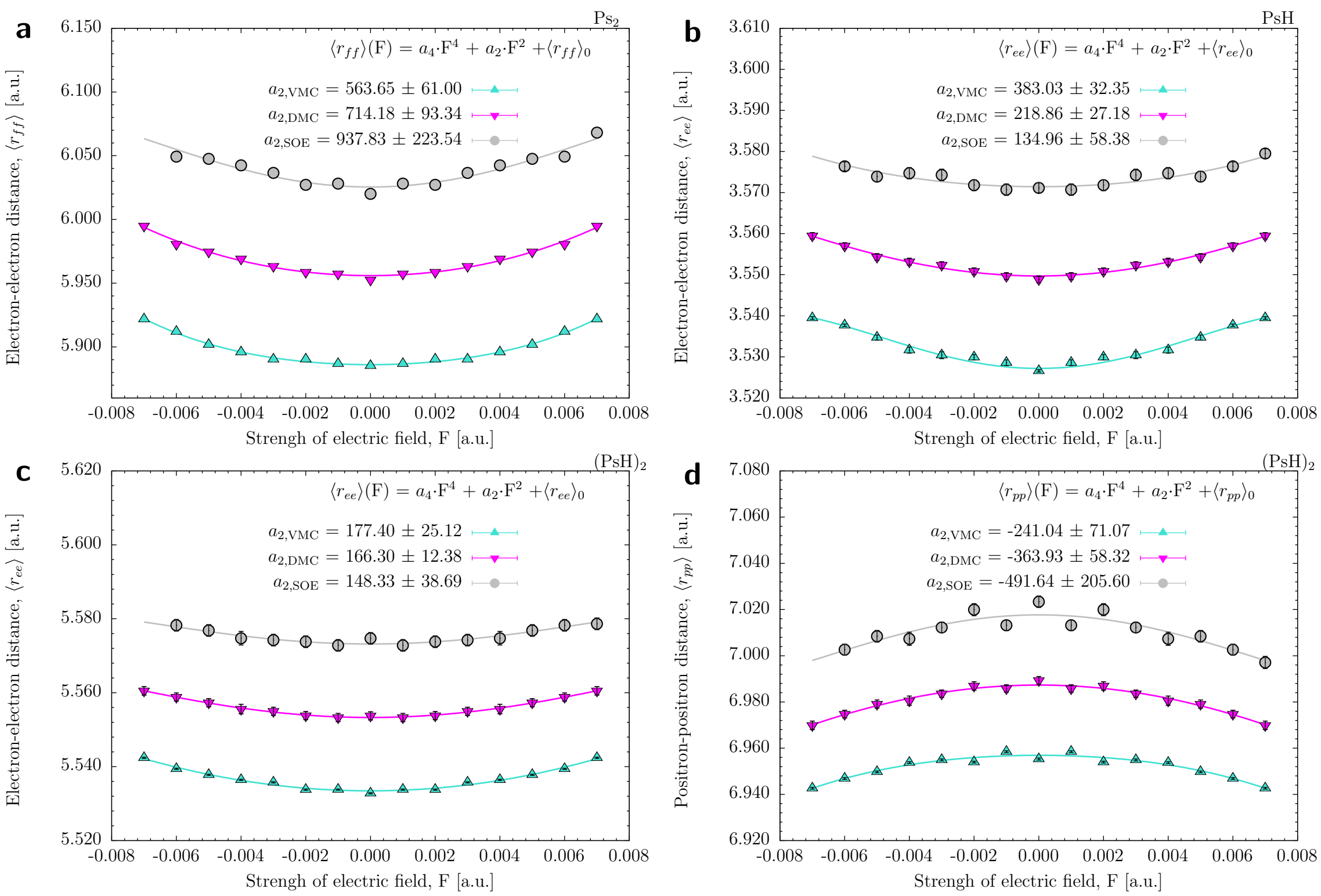

Figure S10: **Fermion-Fermion distances response.**

Polynomial fitting of the expectation values of the same type of fermionic particle distance as a function of the strength of an external electric field, computed with VMC, DMC, and SOE for electron-electron (equal to positron-positron) in positronium dimer: Ps_2 (**a**), electron-electron in positronium hydride: PsH (**b**), electron-electron (**c**) and positron-positron (**d**) in positronium hydride dimer: $(PsH)_2$, in the parallel component.

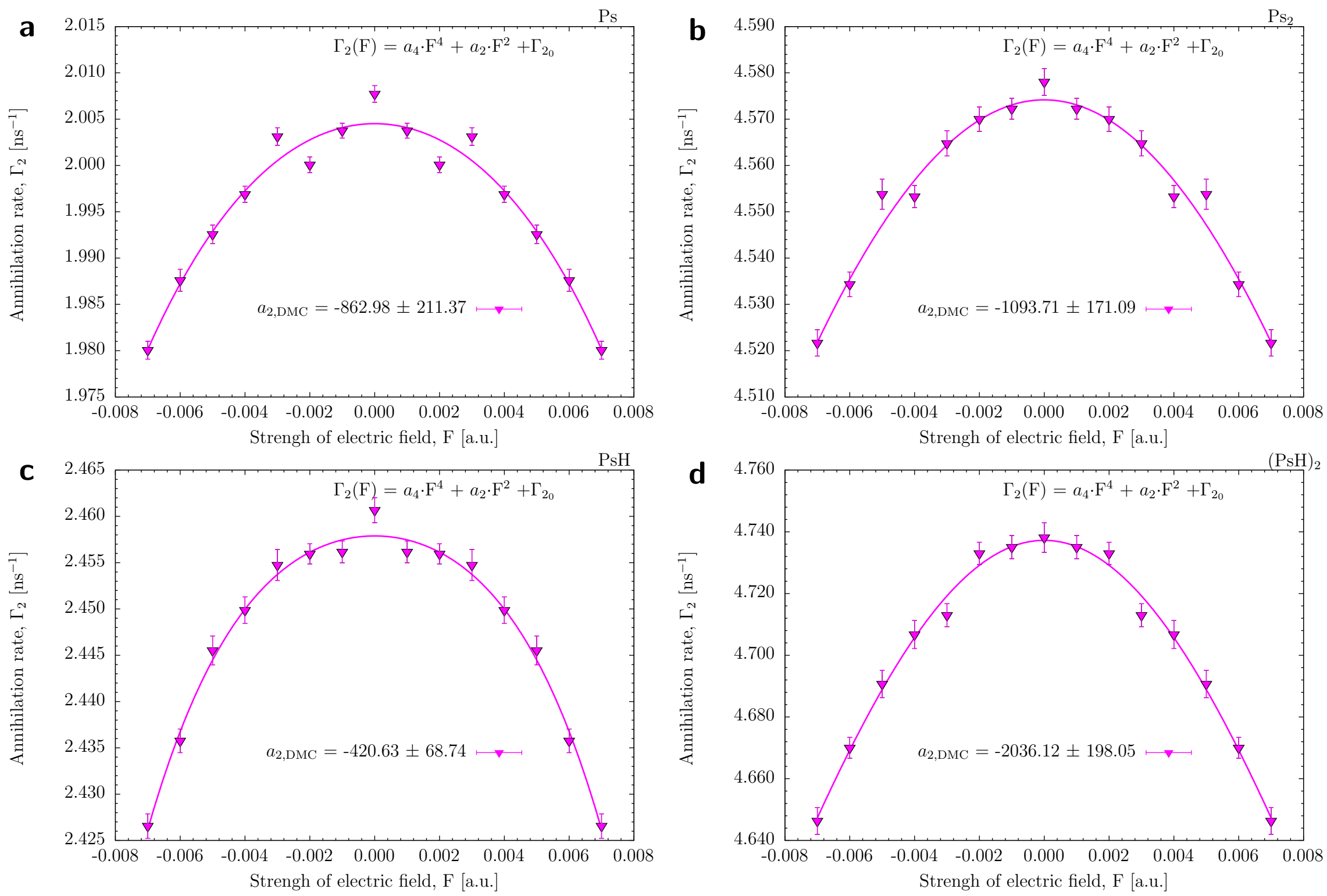

Figure S11: **Annihilation rates response.**

Polynomial fitting of the electron-positron pair annihilation rates as a function of the strength of an external electric field, computed with DMC for positronium: Ps (**a**), positronium dimer: Ps_2 (**b**), positronium hydride: PsH (**c**), and positronium hydride dimer: $(PsH)_2$ in the parallel component (**d**).

Table S2: **Electric field strength effect on fermionic distances.**

Quadratic fitting coefficients, a_2 of the polynomial $G(F) = a_0 + a_2F^2 + a_4F^4$ for expectation values of the distances $\langle r \rangle$ (in bohr) and annihilation rates (in ns^{-1}) as a function of the strength of the external static potential, F.

	Ps	Ps_2	PsH	$(PsH)_2$
$a_{2,VMC}\langle r_{ee}\rangle$	-	564(61)	383(32)	177(25)
$a_{2,DMC}\langle r_{ee}\rangle$	-	714(93)	219(27)	166(12)
$a_{2,SOE}\langle r_{ee}\rangle$	-	873(211)	125(54)	156(36)
$a_{2,VMC}\langle r_{pp}\rangle$	-	564(61)	-	-241(71)
$a_{2,DMC}\langle r_{pp}\rangle$	-	714(93)	-	-364(58)
$a_{2,SOE}\langle r_{pp}\rangle$	-	873(211)	-	-479(186)
$a_{2,VMC}\langle r_{ep}\rangle$	620(14)	662(33)	820(62)	640(31)
$a_{2,DMC}\langle r_{ep}\rangle$	647(13)	737(48)	657(33)	634(20)
$a_{2,SOE}\langle r_{ep}\rangle$	677(31)	817(110)	644(33)	632(64)
$a_{2,DMC}\Gamma_2$	-431(106)	-1094(171)	-421(69)	-2036(198)

10 Benchmark on response properties

In Tab. S3 we benchmark our approach by comparing the Variational Monte Carlo (VMC) and Diffusion Monte Carlo (DMC) for the energies and the Second Order Estimator (SOE), $\langle\hat{A}\rangle_{\mathrm{SOE}} \approx 2\langle\hat{A}\rangle_{\mathrm{DMC}} - \langle\hat{A}\rangle_{\mathrm{VMC}}$, for the electronic and positronic properties, with the results present in the literature.

First, we can see that from the results reported in Tab. S3, the DMC energies are all in agreement with the reference values, within the error bars, while all the expectation values of the interparticle distances, and annihilation rates are in agreement with the references within less than 1% relative error. The least accurate estimations are those obtained for the annihilation rates Γ_2 of Ps_2, which have a relative error of 0.9%, due to the larger error in the VMC estimation that greatly influences the SOE (see SM).

Regarding the average interparticle distances $\langle r_{ff}\rangle$(F) and the annihilation rates Γ_2 in Tab. S3, we also report in Tab. S2 the non-zero second-degree fitting coefficient a_2 used to estimate the zero field quantities

Such coefficients characterize the variation of the quantities with respect to the external potential, with positive values indicating an increment as a function of the increase of the field's intensity, and negative values indicating the opposite.

In general, the increase of the external field has the effect of increasing the average distances between all the particles, as can be seen for the case of Ps, Ps_2 and PsH, and decreasing the annihilation rates Γ_2, since the increase of $\langle r_{ep}\rangle$(F) induces of reduction of the contact density between the positrons and the electrons. More specifically, a field of 0.007 a.u. induces a mere change of just 0.005 to 0.05 bohr in the expectation values of the distance, keeping almost unchanged the internal structure of the quantum systems, and a 1~2% change in the annihilation rates, which corresponds to a 2~5 picoseconds increase in the metastable states' lifetimes.

A different behaviour can be seen only in the value of a_2 for the $\langle r_{pp}\rangle$(F) distance of the $(PsH)_2$ molecule, which is negative, meaning that the distance between the pairs of positron molecule diminishes as a function of the increase of the field's intensity. This is due to the particular bond that is formed between the two positrons that mediate the interaction between the two H^- ions.

In Tab. S3 we report the polarizability of the prototypical conformers. These are obtained as

Table S3: **Benchmark molecular properties of Ps, Ps_2, PsH, and $(PsH)_2$.**

Dipole polarizabilities (a.u.), expectation values of Interparticle distances (bohr), and two-photon annihilation rates (ns^{-1}) for Ps, Ps_2, PsH and $(PsH)_2$ systems computed at VMC and DMC levels.

	**Ps**	**Ps_2**	**PsH**	**$(PsH)_2$**
E_{VMC}	-0.24999997(1)	-0.51487(1)	-0.788481(3)	-1.581927(8)
E_{DMC}	-0.2500000(1)	-0.516007(3)	-0.789199(2)	-1.58864(1)
E_{ref}	-0.25^{a}	-0.516004^{b}	$-0.789196710(4)^{b}$	$-1.5888(1)^{c}$
$\langle r_{ee} \rangle_{VMC}$	-	5.8852(7)	3.5266(3)	5.5328(2)
$\langle r_{ee} \rangle_{DMC}$	-	5.953(1)	3.5489(8)	5.554(1)
$\langle r_{ee} \rangle_{SOE}$	-	6.020(2)	3.571(1)	5.575(2)
$\langle r_{ee} \rangle_{ref}$	-	6.03321	3.574788	-
$\langle r_{pp} \rangle_{VMC}$	-	5.8852(7)	-	6.9553(4)
$\langle r_{pp} \rangle_{DMC}$	-	5.953(1)	-	6.989(2)
$\langle r_{pp} \rangle_{SOE}$	-	6.020(2)	-	7.023(2)
$\langle r_{pp} \rangle_{ref}$	-	6.03321	-	-
$\langle r_{ep} \rangle_{VMC}$	3.0008(2)	4.4161(4)	3.4625(2)	5.1197(2)
$\langle r_{ep} \rangle_{DMC}$	3.0010(7)	4.4481(9)	3.4705(7)	5.133(1)
$\langle r_{ep} \rangle_{SOE}$	3.0012(10)	4.480(1)	3.478(1)	5.146(2)
$\langle r_{ep} \rangle_{ref}$	3^{a}	4.487155^{b}	3.480273^{b}	-
$\Gamma_{2,VMC}$	2.01(1)	4.73(3)	2.441(6)	4.58(1)
$\Gamma_{2,DMC}$	2.0077(9)	4.578(3)	2.461(1)	4.738(5)
$\Gamma_{2,SOE}$	2.00(1)	4.42(3)	2.480(7)	4.89(1)
$\Gamma_{2,ref}$	2.008^{a}	4.465106^{b}	2.471406^{b}	-
α_{VMC} [E]	35.65(2)	73.5(5)	43.5(9)	133.4(9)
α_{DMC} [E]	35.99(1)	71.6(2)	42.4(3)	129(1)
α_{VMC} [μ]	33.5(1)	71.0(1)	38(2)	133.2(3)
α_{DMC} [μ]	34.74(8)	71.3(1)	39.4(5)	131.8(2)
α_{SOE} [μ]	36.0(1)	71.7(2)	42.6(1)	130.5(2)
α_{ref}	36^{d}	$71.7(2)^{d}$	$42.2836(5)^{e}$	-
α^{e}_{SOE} [μ^{e}]	18.0(1)	35.8(5)	0.3(3)	0.5(2)
α^{p}_{SOE} [μ^{p}]	18.0(1)	35.8(5)	42.3(4)	130.0(2)

[a] Ps analytical values from ref. 81 [b] Explicitly correlated gaussians (ECG) from ref. 82 [c] DMC (*25*) [d] Path Integral Monte Carlo (PIMC) from ref. 78 [e] Hylleraas wave function from ref. 30

both the first-order derivative of the dipole moment (using SOE) and then second order derivatives of the energies (see SM for further details in our recommended extrapolation methodology). Importantly, the two estimations coincide within the error bars and with the accurate reference calculations present in the literature (*30, 78*).

For Ps_2, the results confirm the field-free Path-Integral Monte Carlo (PIMC) reference value in ref. 78, which shows that the system has a slightly reduced polarizability compared to the two separate Ps, meaning that its polarizability is less than $2\alpha_{\mathrm{Ps}}$, despite the large increase in the expected electron-positron average distance. For PsH, the estimated value of the polarizability is close to the highly accurate and precise value of Yan *et al.* obtained with extrapolated Hylleraas functions (*30*), corroborating all previous calculations, in contrast to the first reported value of 123 a.u. (*83*).

11 Potential energy curves and polarizability curves

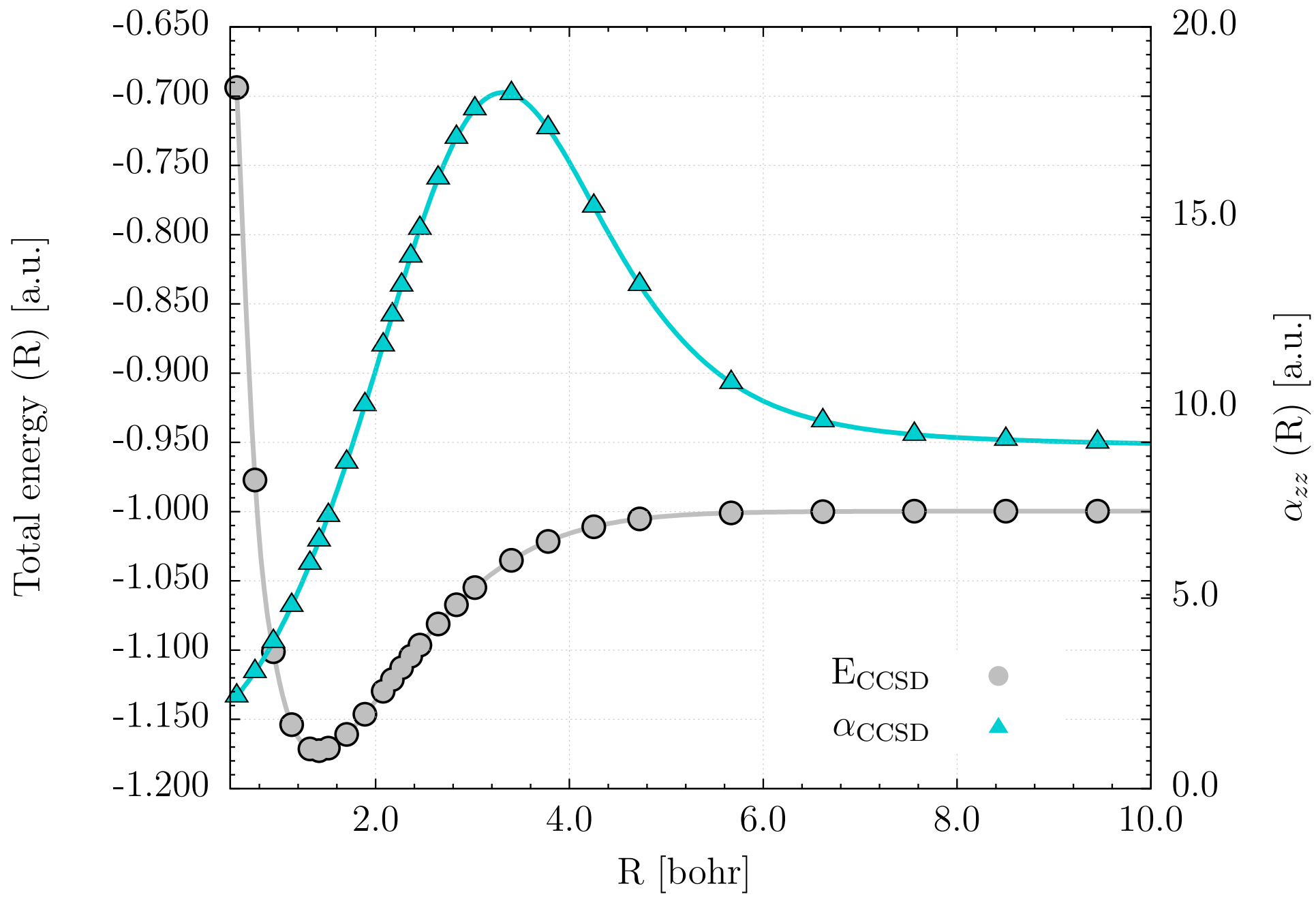

Figure S12: **Evolution of H_2 energy and polarizability with bond distance.**

Total energy and dipole polarizability computed at CCSD/aug-cc-pVTZ level.

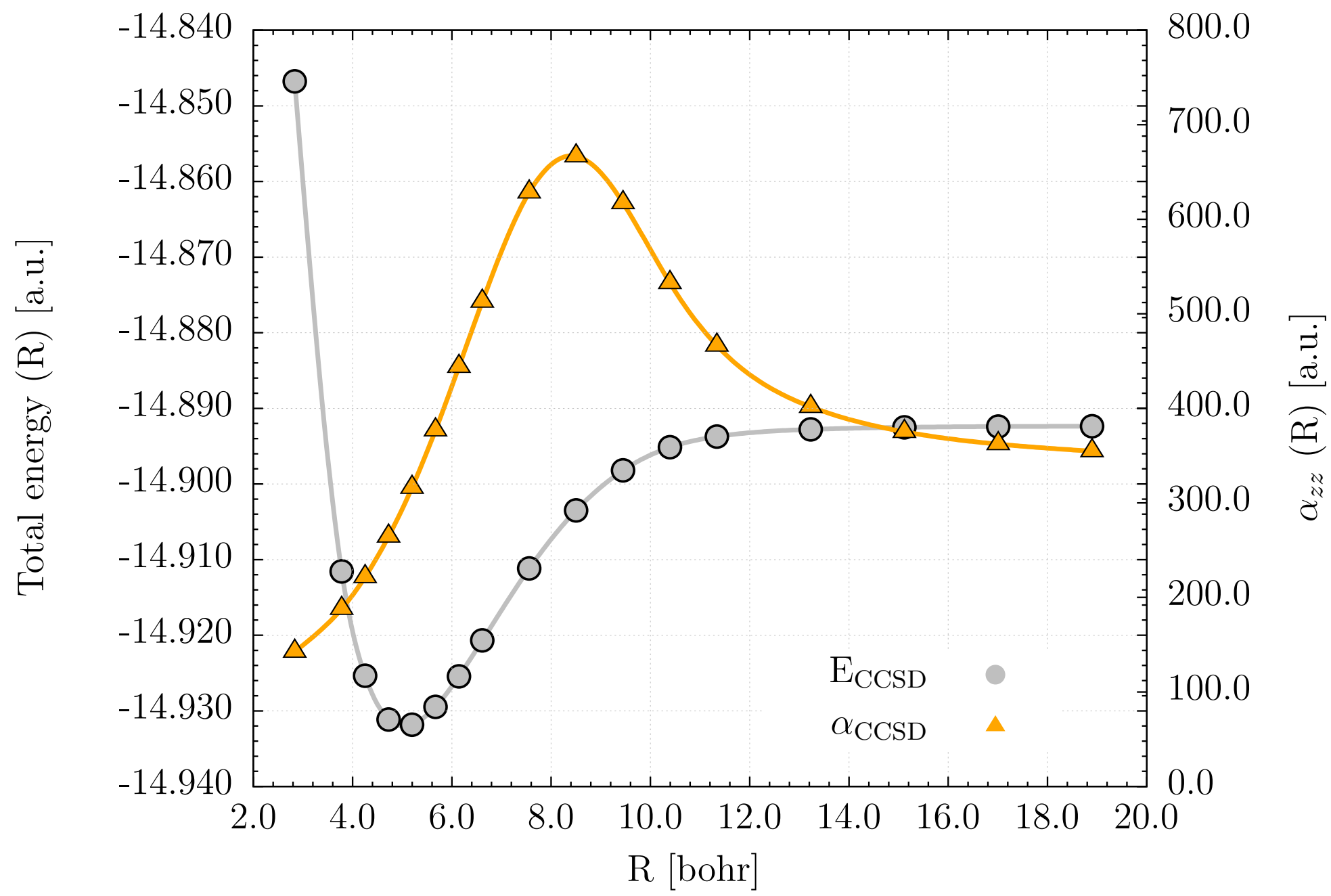

Figure S13: **Evolution of Li_2 energy and polarizability with bond distance.**

Total energy and dipole polarizability computed at CCSD/aug-cc-pVTZ level

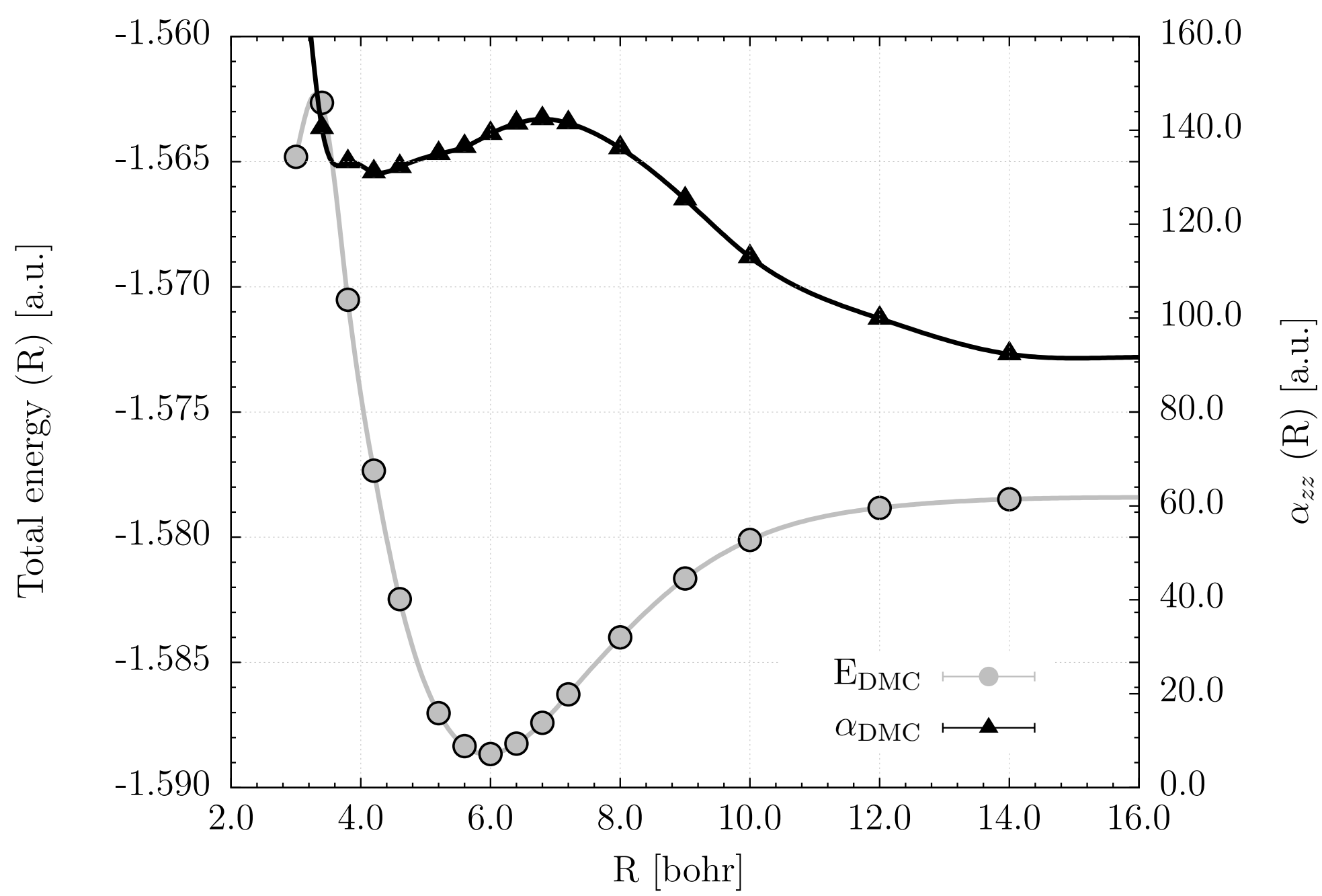

Figure S14: **Evolution of $(PsH)_2$ energy and polarizability with bond distance.**

Total energy and dipole polarizability computed at DMC level with a 0.001 a.u. time step

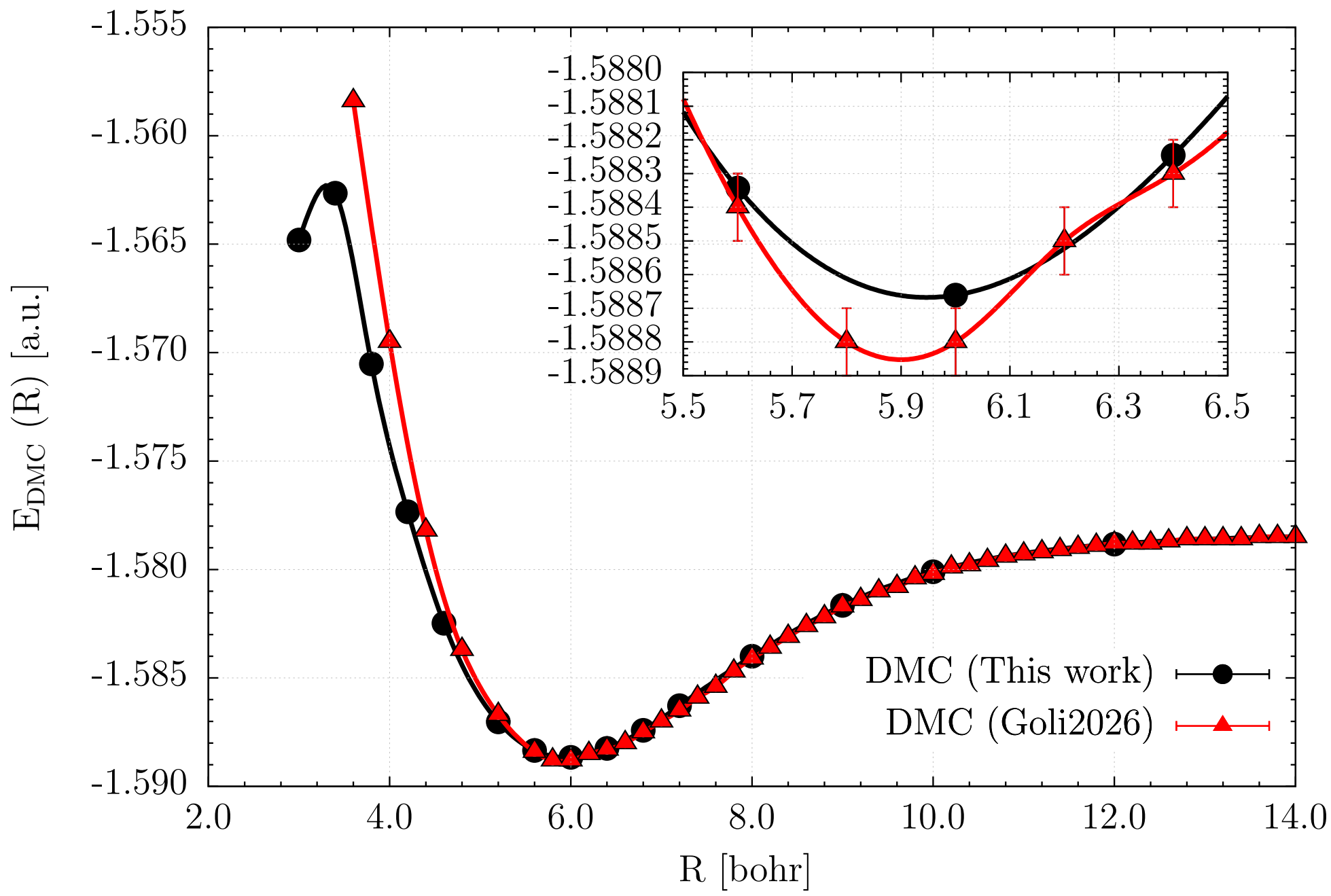

Figure S15: **Potential energy curves comparison for $(PsH)_2$.**

Comparison of the total energies obtained in this work against the Goli2026 data taken from reference (*32*), both computed at DMC level with a 0.001 and 0.003 a.u. time step, respectively.

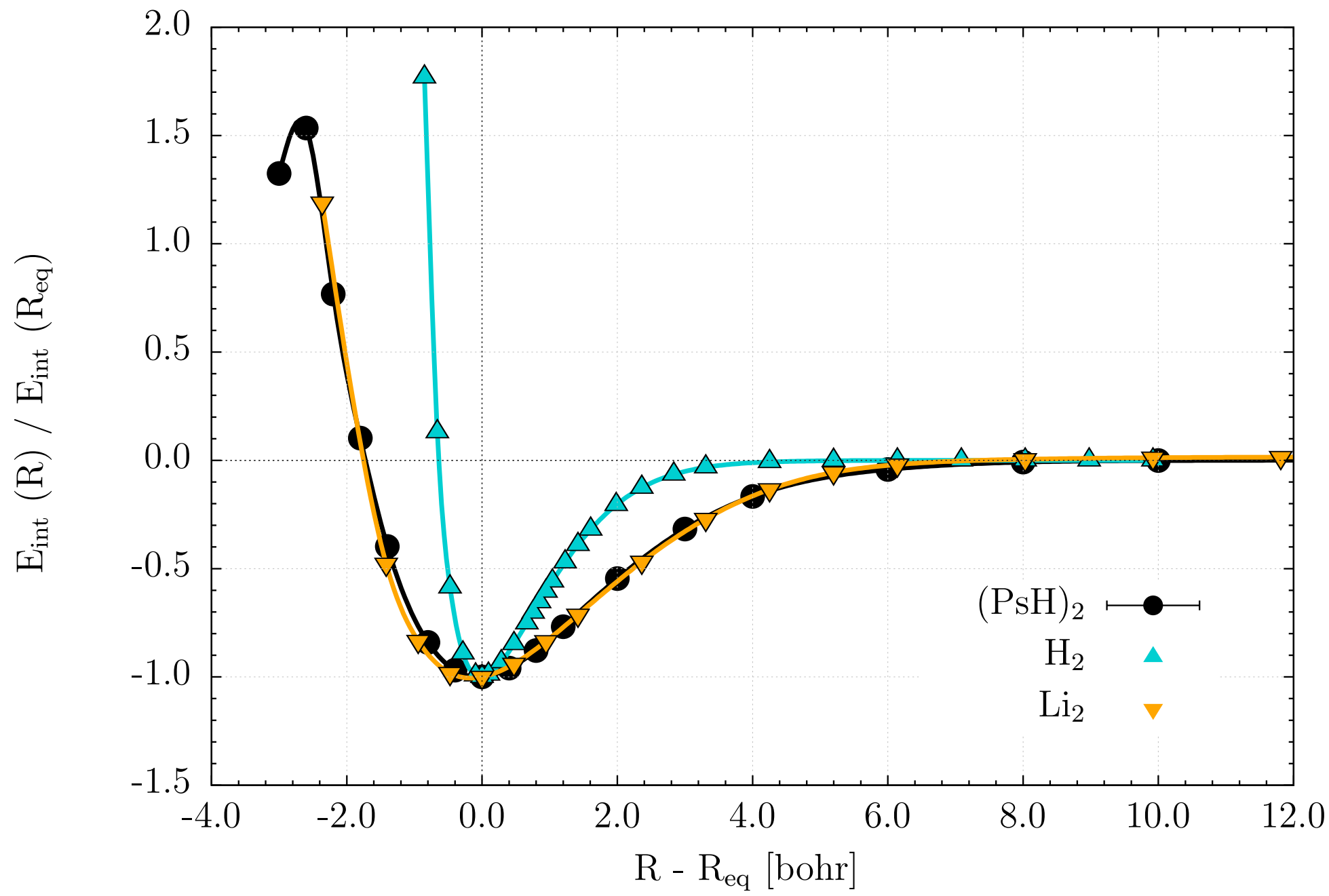

Figure S16: **Evolution of interaction energy with bond distance.**

Relative interaction energies as a function of the internuclear separation shifted to the equilibrium for $(PsH)_2$ (black circles), H_2 (turquoise up-triangles), and Li_2 (orange down-triangles)

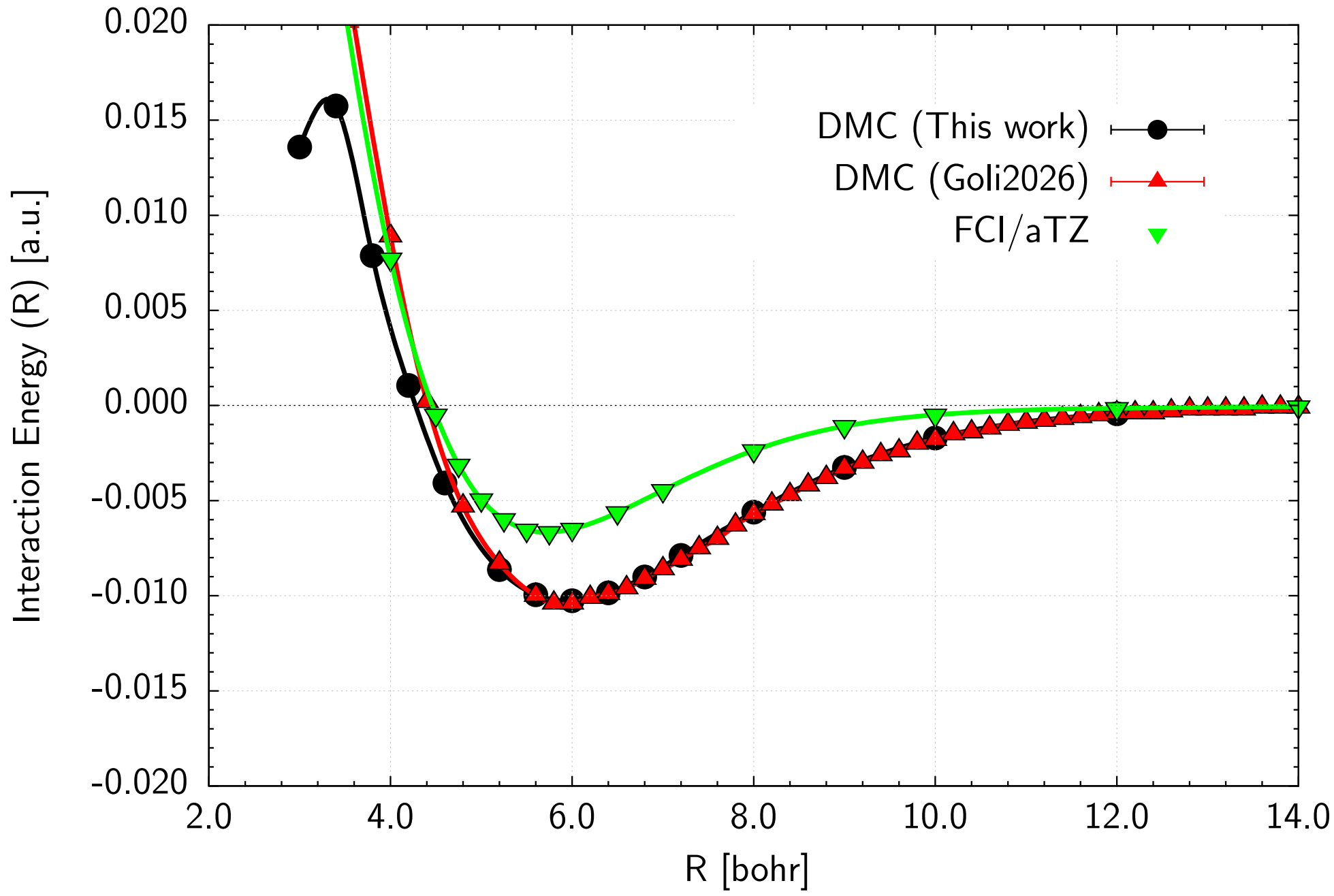

Figure S17: **FCI potential energy curve benchmark for$(PsH)_2$.**

Comparison of the interaction energies at QMC and Full Configuration Interaction (FCI/aug-cc-pVTZ) with a reduced active space selected from the natural orbitals of a preceding CISD calculation.

REFERENCES

1. D. Bödeker, W. Buchmüller, Baryogenesis from the weak scale to the grand unification scale. *Rev. Mod. Phys.* **93**, 035004 (2021).

2. A. Tkatchenko, D. V. Fedorov, Casimir self-interaction energy density of quantum electrodynamic fields. *Phys. Rev. Lett.* **130**, 041601 (2023).

3. S. D. Bass, S. Mariazzi, P. Moskal, E. Stepien, *Colloquium*: Positronium physics and biomedical applications. *Rev. Mod. Phys.* **95**, 021002 (2023).

4. D. W. Gidley, H.-G. Peng, R. S. Vallery, Positron annihilation as a method to characterize porous materials. *Annu. Rev. Mater. Res.* **36**, 49–79 (2006).

5. A. N. Singh, Positron annihilation spectroscopy in tomorrow's material defect studies. *Appl. Spectrosc. Rev.* **51**, 359–378 (2016).

6. C. D. Anderson, The positive electron. *Phys. Rev* **43**, 491–494 (1933).

7. S. Schippers, E. Sokell, F. Aumayr, H. Sadeghpour, K. Ueda, I. Bray, K. Bartschat, A. Murray, J. Tennyson, A. Dorn, M. Yamazaki, M. Takahashi, N. Mason, O. Novotný, A. Wolf, L. Sanche, M. Centurion, Y. Yamazaki, G. Laricchia, C. M. Surko, J. Sullivan, G. Gribakin, D. W. Savin, Y. Ralchenko, R. Hoekstra, G. O'Sullivan, Roadmap on photonic, electronic and atomic collision physics: II. Electron and antimatter interactions. *J. Phys. B* **52**, 171002 (2019).

8. J. R. Danielson, E. Arthur-Baidoo, C. M. Surko, Improved positron-molecule binding energies and estimations using molecular parameters. *Phys. Rev. A* **111**, 042809 (2025).

9. A. Dupasquier, Allen P. Mills, Roberto S. Brusa, Eds., *Physics with Many Positrons* (IOS Press, 2010).

10. P. Moskal, K. Dulski, N. Chug, C. Curceanu, E. Czerwiński, M. Dadgar, J. Gajewski, A. Gajos, G. Grudzień, B. C. Hiesmayr, K. Kacprzak, Ł. Kapłon, H. Karimi, K. Klimaszewski, G. Korcyl, P. Kowalski, T. Kozik, N. Krawczyk, W. Krzemień, E. Kubicz, P. Małczak, S. Niedźwiecki, M. Pawlik-Niedźwiecka, M. Pędziwiatr, L. Raczyński, J. Raj, A. Ruciński, S. Sharma, Shivani, R.

Y. Shopa, M. Silarski, M. Skurzok, E. Ł. Stępień, M. Szczepanek, F. Tayefi, W. Wiślicki, Positronium imaging with the novel multiphoton PET scanner. *Sci. Adv.* **7**, eabh4394 (2021).

11. J. Hofierka, B. Cunningham, C. M. Rawlins, C. H. Patterson, D. G. Green, Many-body theory of positron binding to polyatomic molecules. *Nature* **606**, 688–693 (2022).

12. G. Cassella, W. M. C. Foulkes, D. Pfau, J. S. Spencer, Neural network variational Monte Carlo for positronic chemistry. *Nat. Commun.* **15**, 5214 (2024).

13. S. Upadhyay, A. Benali, K. D. Jordan, Capturing correlation effects in positron binding to atoms and molecules. *J. Chem. Theory Comput.* **20**, 9879–9893 (2024).

14. D. Bressanini, M. Mella, G. Morosi, Positronium chemistry by quantum Monte Carlo. I. Positronium-first row atom complexes. *J. Chem. Phys.* **108**, 4756–4760 (1998).

15. M. Charlton, J. W. Humberston, *Positron Physics* (Cambridge Univ. Press, 2009).

16. Y. C. Jean, P. E. Mallon, D. M. Schrader, *Principles and Applications of Positron and Positronium Chemistry* (World Scientific, 2003).

17. H. Chojnacki, K. Strasburger, "Quantum chemical calculations on positronic systems," in *Explicitly Correlated Wave Functions in Chemistry and Physics* (Springer, 2003), pp. 439–463.

18. G. F. Gribakin, J. A. Young, C. M. Surko, Positron-molecule interactions: Resonant attachment, annihilation, and bound states. *Rev. Mod. Phys.* **82**, 2557–2607 (2010).

19. Y. Kita, M. Tachikawa, "Quantum Monte Carlo study of the binding of a positron to polar molecules," in *Advances in Quantum Monte Carlo* (American Chemical Society, 2012), pp. 157–173.

20. J. Charry, M. T. N. Varella, A. Reyes, Binding matter with antimatter: The covalent positron bond. *Angew. Chem. Int. Ed. Engl.* **57**, 8859–8864 (2018).

21. F. Moncada, L. Pedraza-González, J. Charry, M. T. d. N. Varella, A. Reyes, Covalent bonds in positron dihalides. *Chem. Sci.* **11**, 44–52 (2020).

22. T. Tachibana, D. Hoshi, Y. Nagashima, Molecular ion desorption from LiF (110) surfaces by positron annihilation. *Phys. Rev. Lett.* **131**, 143201 (2023).

23. R. Porras-Roldan, J. Charry, F. Moncada, R. Flores-Moreno, M. T. d. N. Varella, A. Reyes, Watch out electrons!: Positron binding redefines chemical bonding in Be_2. *Chem. Sci.* **16**, 22322–22332 (2025).

24. J. P. Cassidy, J. Hofierka, B. Cunningham, D. G. Green, Many-body theory calculations of positronic-bonded molecular dianions. *J. Chem. Phys.* **160**, 084304 (2024).

25. D. Bressanini, Two positrons can form a chemical bond in (PsH)2. *J. Chem. Phys.* **155**, 054306 (2021).

26. D. Archila-peña, F. Moncada, J. Charry, T. N. Varella, R. Flores-moreno, F. J. Torres, A. Reyes, Two-positron-bonded dihalides: Ps_2XY (X, Y = F, Cl, Br). *Chem. Eur. J.* **30**, 202402618 (2024).

27. J. Charry, F. Moncada, M. Barborini, L. Pedraza-González, M. T. d. N. Varella, A. Tkatchenko, A. Reyes, The three-center two-positron bond. *Chem. Sci.* **13**, 13795–13802 (2022).

28. D. Bressanini, $e^+(PsH)_2$: A three-positron molecule with a positronic chemical bond. *J. Chem. Phys.* **156**, 154302 (2022).

29. M. Mella, D. Bressanini, G. Morosi, Variational Monte Carlo calculation of dynamic multipole polarizabilities and van der Waals coefficients of the PsH system. *Phys. Rev. A* **63**, 024503 (2001).

30. Z.-C. Yan, Polarizabilities and dispersion coefficients of positronium hydride. *J. Phys. B At. Mol. Opt. Phys.* **35**, L345–L349 (2002).

31. M. Goli, D. Bressanini, S. Shahbazian, On the nature of the two-positron bond: Evidence for a novel bond type. *Phys. Chem. Chem. Phys.* **25**, 29531–29547 (2023).

32. M. Goli, D. Bressanini, S. Shahbazian, The two-positron gluic bond as a manifestation of 'super' van der Waals interactions. *Phys. Chem. Chem. Phys* **28**, 11154–11160 (2026).

33. Y. Kita, M. Tachikawa, N. D. Drummond, R. J. Needs, A variational monte carlo study of positronic compounds using inhomogeneous backflow transformations. *Chem. Lett.* **39**, 1136–1137 (2010).

34. J. A. Charry Martinez, M. Barborini, A. Tkatchenko, Correlated wave functions for electron–positron interactions in atoms and molecules. *J. Chem. Theory Comput.* **18**, 2267–2280 (2022).

35. F. Marsusi, E. Mostaani, N. D. Drummond, Quantum Monte Carlo study of three-dimensional Coulomb complexes: Trions and biexcitons, hydrogen molecules and ions, helium hydride cations, and positronic and muonic complexes. *Phys. Rev. A* **106**, 62822 (2022).

36. K. A. Simula, J. E. Muff, I. Makkonen, N. D. Drummond, Quantum Monte Carlo study of positron lifetimes in solids. *Phys. Rev. Lett.* **129**, 166403 (2022).

37. M. Barborini, J. Charry, M. Ditte, A. Leventis, G. Kafanas, A. Tkatchenko, QMeCha: Quantum Monte Carlo package for fermions in embedding environments. *J. Chem. Phys.* **164**, 062501 (2026).

38. J. Charry, M. Barborini, A. Tkatchenko, Unraveling chemical bonding mechanisms through dipole moment variations under external electric fields. *Phys. Chem. Chem. Phys.* **27**, 23044–23055 (2025).

39. V. Postils, M. Garcia-Borràs, M. Solà, J. M. Luis, E. Matito, On the existence and characterization of molecular electrides. *Chem. Commun.* **51**, 4865–4868 (2015).

40. S. P. Sitkiewicz, E. Ramos-Cordoba, J. M. Luis, E. Matito, How many electrons does a molecular electride hold? *J. Phys. Chem. A* **125**, 4819–4835 (2021).

41. J. G. Ángyán, E. Rosta, P. R. Surján, Covalent bond orders and atomic valences from correlated wavefunctions. *Chem. Phys. Lett.* **299**, 1–8 (1999).

42. C. Outeiral, M. A. Vincent, Á. M. Pendás, P. L. A. Popelier, Revitalizing the concept of bond order through delocalization measures in real space. *Chem. Sci.* **9**, 5517–5529 (2018).

43. A. F. Silva, L. J. Duarte, P. L. Popelier, Contributions of IQA electron correlation in understanding the chemical bond and non-covalent interactions. *Struct. Chem.* **31**, 507–519 (2020).

44. M. Bollhöfer, Y. Notay, JADAMILU: A software code for computing selected eigenvalues of large sparse symmetric matrices. *Comput. Phys. Commun.* **177**, 951–964 (2007).

45. R. Flores-Moreno, E. Posada, F. Moncada, J. Romero, J. Charry, M. Díaz-Tinoco, S. A. González, N. F. Aguirre, A. Reyes, LOWDIN: The any particle molecular orbital code. *Int. J. Quantum Chem.* **114**, 50–56 (2014).

46. M. García-Revilla, P. L. Popelier, E. Francisco, Á. Martín Pendás, Nature of chemical interactions from the profiles of electron delocalization indices. *J. Chem. Theory Comput.* **7**, 1704–1711 (2011).

47. D. Hait, M. Head-Gordon, When is a bond broken? The polarizability perspective. *Angew. Chem. Int. Ed. Engl.* **62**, e202312078 (2023).

48. A. J. Sterling, D. S. Levine, A. Aldossary, M. Head-Gordon, Chemical bonding and the role of node-induced electron confinement. *J. Am. Chem. Soc.* **146**, 9532–9543 (2024).

49. J. Rychlewski, An accurate calculation of the polarizability of the hydrogen molecule and its dependence on rotation, vibration and isotopic substitution. *Mol. Phys.* **41**, 833–842 (1980).

50. G. Gopakumar, M. Abe, M. Hada, M. Kajita, Dipole polarizability of alkali-metal (Na, K, Rb)-alkaline-earth-metal (Ca, Sr) polar molecules: Prospects for alignment. *J. Chem. Phys* **140**, 224303 (2014).

51. E. Miliordos, K. L. Hunt, Dependence of the multipole moments, static polarizabilities, and static hyperpolarizabilities of the hydrogen molecule on the H-H separation in the ground singlet state. *J. Chem. Phys.* **149**, 234103 (2018).

52. D. Bressanini, G. Morosi, Compact boundary-condition-determined wave function for positronium hydride (PsH). *J. Chem. Phys* **119**, 7037–7042 (2003).

53. Y. Toyama, T. Azuma, D. A. Bennett, W. B. Doriese, M. S. Durkin, J. W. Fowler, J. D. Gard, T. Hashimoto, R. Hayakawa, Y. Ichinohe, K. Ishida, S. Kanda, N. Kawamura, Y. Kino, R. Konishi, Y. Miyake, K. M. Morgan, R. Nakashima, H. Natori, H. Noda, G. C. O'Neil, S. Okada, T. Okumura, K. Okutsu, C. D. Reintsema, K. Sasaki, T. Sato, D. R. Schmidt, K. Shimomura, P. Strasser, D. S. Swetz, T. Takahashi, M. Tampo, H. Tatsuno, J. N. Ullom, I. Umegaki, S. Watanabe, S. Yamada, T. Yamashita, Direct observation of muonic molecules in resonance states critical to muon catalyzed fusion. *Sci. Adv.* **12**, eaed3321 (2026).

54. D. G. Fleming, J. Manz, K. Sato, T. Takayanagi, Fundamental change in the nature of chemical bonding by isotopic substitution. *Angew. Chem., Int. Ed. Engl.* **53**, 13706–13709 (2014).

55. D. G. A. Smith, L. A. Burns, A. C. Simmonett, R. M. Parrish, M. C. Schieber, R. Galvelis, P. Kraus, H. Kruse, R. Di Remigio, A. Alenaizan, A. M. James, S. Lehtola, J. P. Misiewicz, M. Scheurer, R. A. Shaw, J. B. Schriber, Y. Xie, Z. L. Glick, D. A. Sirianni, J. S. O'Brien, J. M. Waldrop, A. Kumar, E. G. Hohenstein, B. P. Pritchard, B. R. Brooks, H. F. Schaefer III, A. Y. Sokolov, K. Patkowski, A. E. DePrince III, U. Bozkaya, R. A. King, F. A. Evangelista, J. M. Turney, T. D. Crawford, C. D. Sherrill, Psi4 1.4: Open-source software for high-throughput quantum chemistry. *J. Chem. Phys.* **152**, 184108 (2020).

56. S. Varrette, P. Bouvry, H. Cartiaux, F. Georgatos, Management of an academic HPC Cluster: The UL experience, in *Proceedings of the 2014 International Conference on High Performance Computing and Simulation (HPCS 2014)* (IEEE, 2014), pp. 959–967.

57. J. Jiang, J. Mitroy, Y. Cheng, M. W. J. Bromley, Effective oscillator strength distributions of spherically symmetric atoms for calculating polarizabilities and long-range atom–atom interactions. *At. Data Nucl. Data Tables* **101**, 158–186 (2015).

58. W. M. C. Foulkes, L. Mitas, R. J. Needs, G. Rajagopal, Quantum Monte Carlo simulations of solids. *Rev. Mod. Phys* **73**, 33–83 (2001).

59. M. H. Kalos, P. A. Whitlock, “Quantum Monte Carlo,” in *Monte Carlo Methods* (John Wiley & Sons, Ltd., 2000), pp. 159–178.

60. F. Becca, S. Sorella, *Quantum Monte Carlo Approaches for Correlated Systems* (Cambridge Univ. Press, 2017).

61. S. Sorella, Generalized Lanczos algorithm for variational quantum Monte Carlo. *Phys. Rev. B.* **64**, 024512 (2001).

62. S. Sorella, Wave function optimization in the variational Monte Carlo method. *Phys. Rev. B.* **71**, 241103 (2005).

63. C. Filippi, C. Umrigar, Correlated sampling in quantum Monte Carlo: A route to forces. *Phys. Rev. B* **61**, R16291–R16294 (2000).

64. P. J. Reynolds, D. M. Ceperley, B. J. Alder, W. A. Lester, Fixed-node quantum Monte Carlo for molecules. *J. Chem. Phys.* **77**, 5593–5603 (1982).

65. I. Kosztin, B. Faber, K. Schulten, Introduction to the diffusion Monte Carlo method. *Am. J. Phys.* **64**, 633–644 (1996).

66. M. P. Nightingale, C. J. Umrigar, *Quantum Monte Carlo Methods in Physics and Chemistry*, vol. 525 of *NATO Science Series C* (Kluwer Academic Publishers, published in cooperation with NATO Scientific Affairs Division, 1999).

67. C. J. Umrigar, M. P. Nightingale, K. J. Runge, A diffusion Monte Carlo algorithm with very small time-step errors. *J. Chem. Phys.* **99**, 2865–2890 (1993).

68. T. A. Anderson, M. C. Per, C. J. Umrigar, Reducing the time-step errors in diffusion Monte Carlo. *J. Chem. Phys.* **160**, 104110 (2024).

69. F. Della Pia, B. X. Shi, Y. S. Al-Hamdani, D. Alfé, T. A. Anderson, M. Barborini, A. Benali, M. Casula, N. D. Drummond, M. Dubecký, C. Filippi, P. R. C. Kent, J. T. Krogel, P. López Ríos, A. Lüchow, Y. Luo, A. Michaelides, L. Mitas, K. Nakano, R. J. Needs, M. C. Per, A. Scemama,

J. Schultze, R. Shinde, E. Slootman, S. Sorella, A. Tkatchenko, M. Towler, C. J. Umrigar, L. K. Wagner, W. A. Wheeler, H. Zhou, A. Zen, Reproducibility of fixed-node diffusion Monte Carlo across diverse community codes: The case of water–methane dimer. *J. Chem. Phys.* **163**, 104110 (2025).

70. M. D. Towler, The quantum Monte Carlo method. *Phys. Status Solidi B* **243**, 2573–2598 (2006).

71. A. D. Buckingham, Permanent and induced molecular moments and long-range intermolecular forces. *Adv. Chem. Phys* **12**, 107–142 (1967).

72. D. Bressanini, M. Mella, G. Morosi, Stability and positron annihilation of positronium hydride $L = 0,1,2$ states: A quantum Monte Carlo study. *Phys. Rev. A* **57**, 1678–1685 (1998).

73. M. Casula, C. Attaccalite, S. Sorella, Correlated geminal wave function for molecules: An efficient resonating valence bond approach. *J. Chem. Phys.* **121**, 7110–7126 (2004).

74. S. F. Boys, N. C. Handy, J. W. Linnett, A calculation for the energies and wavefunctions for states of neon with full electronic correlation accuracy. *Proc. Math. Phys. Eng. Sci.* **310**, 63–78 (1969).

75. N. D. Drummond, M. D. Towler, R. J. Needs, Jastrow correlation factor for atoms, molecules, and solids. *Phys. Rev. B* **70**, 235119 (2004).

76. D. Bressanini, The stability of $e^+(H^-)_2$. *J. Chem. Phys.* **154**, 224306 (2021).

77. C. Swalina, M. V. Pak, S. Hammes-Schiffer, Analysis of electron-positron wavefunctions in the nuclear-electronic orbital framework. *J. Chem. Phys.* **136**, 164105 (2012).

78. J. Tiihonen, I. Kylänpää, T. T. Rantala, Computation of dynamic polarizabilities and van der waals coefficients from path-integral monte carlo. *J. Chem. Theory Comput.* **14**, 5750–5763 (2018).

79. R. A. Ferrell, Theory of positron annihilation in solids. *Rev. Mod. Phys.* **28**, 308–337 (1956).

80. N. Jiang, D. M. Schrader, Diffusion quantum Monte Carlo calculation of the binding energy and annihilation rate of positronium hydride, PsH, *J. Chem. Phys.* **109**, 9430–9433 (1998).

81. J. Mitroy, M. W. J. Bromley, G. G. Ryzhikh, “Positronic atoms,” in *New Directions in Antimatter Chemistry and Physics*, C. M. Surko, F. A. Gianturco, Eds. (Kluwer Academic Publishers, 2001), pp. 199–221.

82. S. Bubin, L. Adamowicz, Nonrelativistic variational calculations of the positronium molecule and the positronium hydride. *Phys. Rev. A* **74**, 052502 (2006).

83. C. Le Sech, B. Silvi, Study of positronium hydride with a simple wavefunction: Application to the Stark effect of PsH. *Chem. Phys.* **236**, 77–85 (1998).
